# Proglucagon-Derived Peptides as Therapeutics

**DOI:** 10.3389/fendo.2021.689678

**Published:** 2021-05-18

**Authors:** Ryan A. Lafferty, Finbarr P. M. O’Harte, Nigel Irwin, Victor A. Gault, Peter R. Flatt

**Affiliations:** School of Biomedical Sciences, Ulster University, Coleraine, United Kingdom

**Keywords:** proglucagon, glucagon, GLP-1, GLP-2, oxyntomodulin, diabetes, obesity, multi-agonist

## Abstract

Initially discovered as an impurity in insulin preparations, our understanding of the hyperglycaemic hormone glucagon has evolved markedly over subsequent decades. With description of the precursor proglucagon, we now appreciate that glucagon was just the first proglucagon-derived peptide (PGDP) to be characterised. Other bioactive members of the PGDP family include glucagon-like peptides -1 and -2 (GLP-1 and GLP-2), oxyntomodulin (OXM), glicentin and glicentin-related pancreatic peptide (GRPP), with these being produced *via* tissue-specific processing of proglucagon by the prohormone convertase (PC) enzymes, PC1/3 and PC2. PGDP peptides exert unique physiological effects that influence metabolism and energy regulation, which has witnessed several of them exploited in the form of long-acting, enzymatically resistant analogues for treatment of various pathologies. As such, intramuscular glucagon is well established in rescue of hypoglycaemia, while GLP-2 analogues are indicated in the management of short bowel syndrome. Furthermore, since approval of the first GLP-1 mimetic for the management of Type 2 diabetes mellitus (T2DM) in 2005, GLP-1 therapeutics have become a mainstay of T2DM management due to multifaceted and sustainable improvements in glycaemia, appetite control and weight loss. More recently, longer-acting PGDP therapeutics have been developed, while newfound benefits on cardioprotection, bone health, renal and liver function and cognition have been uncovered. In the present article, we discuss the physiology of PGDP peptides and their therapeutic applications, with a focus on successful design of analogues including dual and triple PGDP receptor agonists currently in clinical development.

## Introduction

While the gut hormones secretin and gastrin were discovered almost two decades earlier (1, 2), it was the extraction, isolation and purification of insulin from canine pancreatic extracts in Toronto in 1921, that truly signifies the advent of peptide-based therapeutics (3). Indeed, the first clinical use of animal-derived insulin began the following year. Continued innovation has led to the production of longer-acting formulations (4), as well as biosynthetic, recombinant DNA human insulins in the 1980’s (5). In this respect, it is incredible to think that a century later, insulin remains a vital mainstay in the management of Type 1 diabetes mellitus (T1DM).

Although insulin therapy is often indicated in poorly controlled Type 2 diabetes mellitus (T2DM), this condition is more often managed with diet plus an array of medications that augment remaining endogenous insulin production and function. Indeed, peptide-based therapeutics have become important tools in the management of T2DM, emulating the success of insulin in T1DM. In particular, enzymatically stable analogues, based on the endogenous incretin hormone glucagon-like peptide 1 (GLP-1), are now widely prescribed second- and third-line agents for T2DM (6). Furthermore, orally-available inhibitors of the enzyme dipeptidyl peptidase-4 (DPP-4), which degrades incretins including GLP-1, have been increasingly prescribed since their approval in 2007 (7).

## Proglucagon – Discovery and Processing

As its name suggests, GLP-1 is related to the glucose-elevating hormone, glucagon. Indeed, a family of glucagon-related peptides exists, all of which are derived from differential processing of a common prohormone, proglucagon (8). Whilst glucagon and its hyperglycaemic actions were discovered in 1922 (9), its amino acid sequence was not elucidated until 1957 (10). Furthermore, proglucagon went undiscovered until the early 1980’s, when its cDNA was initially identified in anglerfish (11, 12), with discovery of a proglucagon equivalent in rat (13, 14), hamster (15), cow and human several years later (16). These discoveries were made possible with the advent of lab-scale cDNA cloning techniques, which made it feasible to accurately predict amino acid sequences of proteins by decoding the nucleotide sequences of cloned recombinant cDNA copies of mRNAs. Such experiments highlighted that glucagon and several peptides with a high degree of sequence homology were encoded by this prohormone (11, 12).

Interestingly, anglerfish islets were demonstrated to express two separate proglucagon peptides, meaning a hybrid approach was taken to identify cDNA encoding the 29 amino acid (aa), anglerfish glucagon (11, 12). From there, cDNA encoding for previously sequenced proteins, glicentin and oxyntomodulin was uncovered (17, 18), with glucagon located within the middle portion of this sequence (11). However, the proposed proglucagon sequence exhibited unexpected C-terminal elongation, containing an additional 34-residue glucagon-related carboxyl-terminal peptide, which exhibited structural similarity with another previously sequenced hormone, glucose-dependent insulinotropic polypeptide (GIP) (11, 19). Further study of anglerfish proglucagon led to the characterisation of a second proglucagon cDNA, derived from a different mRNA and gene which encoded glucagon. This shared significant homology with mammalian glucagons, but also a second C-terminal glucagon-related peptide, again comprised of 34 residues with significant sequence homology to glucagon (12).

Whilst work in anglerfish provided an excellent starting point, particularly in highlighting the presence of these carboxy glucagon-related peptides (11, 12), it was the elucidation of the structure of mammalian proglucagon which truly sparked interest in proglucagon-derived peptides (PGDP’s). While sequence homology with anglerfish proglucagon was high, isolation of the first mammalian proglucagon from hamster unveiled organisational differences, with the 158 amino-acid mammalian precursor containing three PGDP arranged in tandem, namely glucagon and what the authors termed, glucagon-like peptides 1 and 2 (GLP-1 and GLP-2) (15). The biological importance of these carboxy-peptides was initially unclear. Through a combined approach employing immunoassays, immunohistochemistry and chromatography of tissue extracts, it was established that GLP-1 and GLP-2 coexisted with glucagon in pancreatic islet cells and with oxyntomodulin in intestinal L-cells, where they are present at vastly greater concentrations than islets (20).

**Figure 1:**
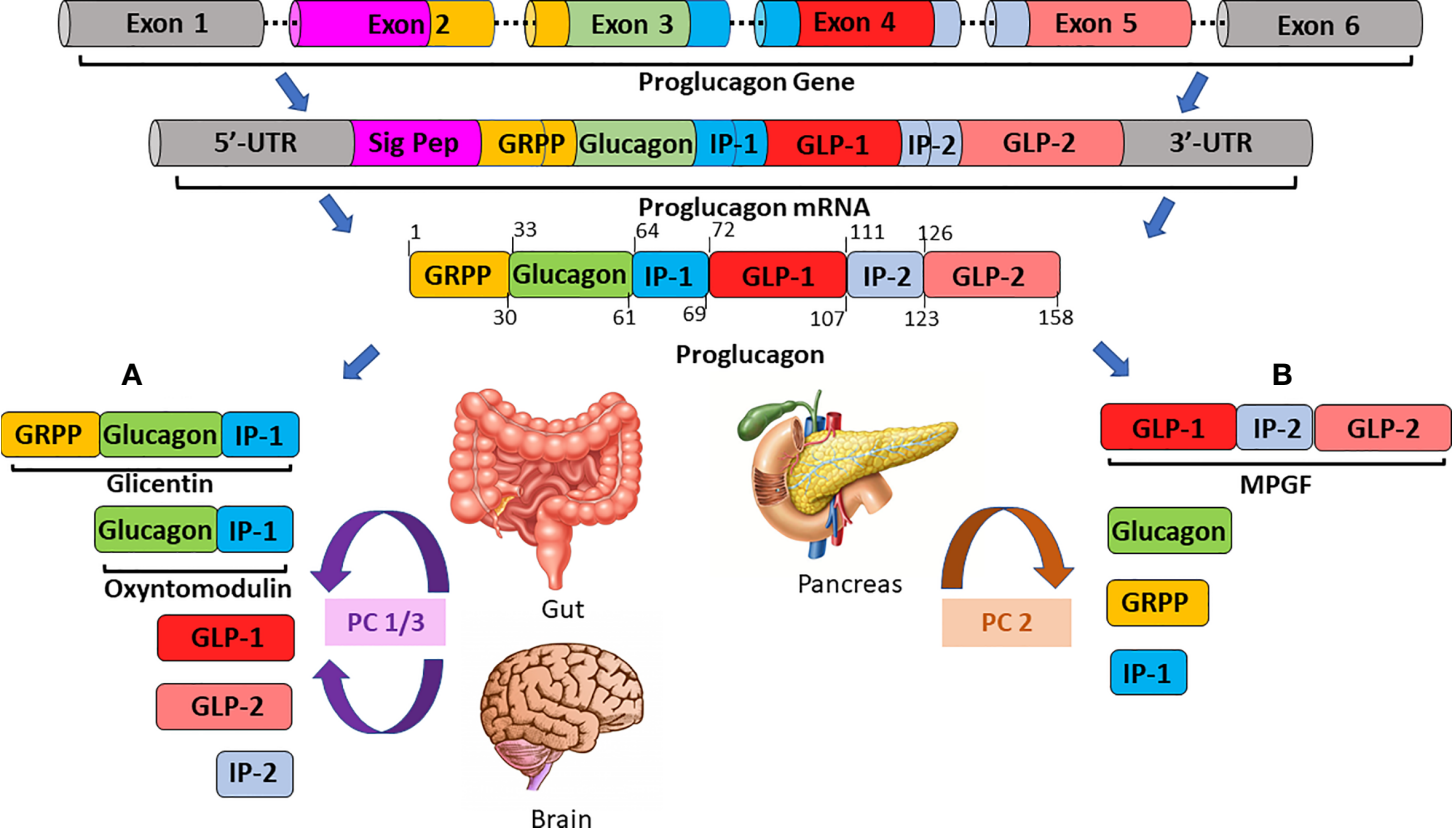
A schematic overview of tissue-specific proglucagon processing in the gut/brain **(A)** and in the pancreas **(B)**. The proglucagon gene, located on chromosome 2 and comprised of 6 exons, is transcribed to generate proglucagon messenger RNA (mRNA). Proglucagon mRNA is subsequently translated to yield the 158 residue, precursor protein, proglucagon. In enteroendocrine L-cells of the ileum and colon **(A)** proglucagon is processed by prohormone convertase 1/3 (PC1/3) to generate glicentin, oxyntomodulin, glucagon-like peptides-1 and -2 (GLP-1, GLP-2) and intervening peptide-2 (IP-2). Conversely, in pancreatic alpha-cells **(B)**, post-translational modification by prohormone convertase 2 (PC2) is responsible for the generation of the major proglucagon fragment (MPGF), glucagon, glicentin-related pancreatic polypeptide (GRPP) and intervening peptide-1 (IP-1).

**Figure 2:**
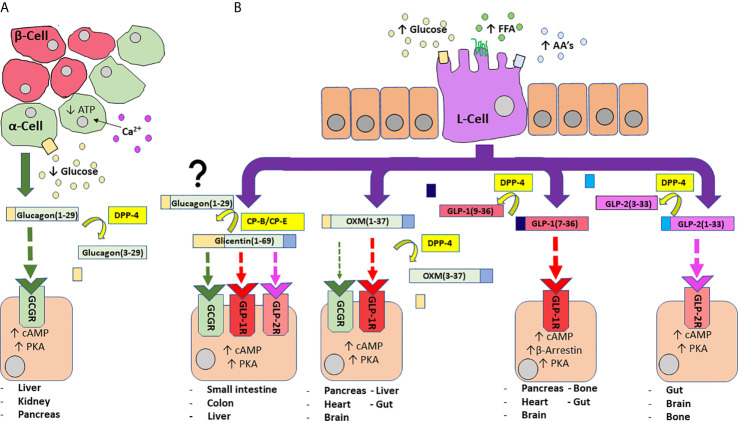
An overview of PGDP actions and secretion from pancreatic alpha-cells **(A)** and enteroendocrine L-cells **(B)**). A fall in circulating glucose concentration sees a reduction in intracellular adenosine triphosphate (ATP) levels and resultant closure of ATP-sensitive K^+^ channels to depolarise the plasma membrane and trigger the influx of Ca^2+^ ions, the primary stimulus for glucagon release **(A)**. Glucagon is subject to N-terminal dipeptide removal by dipeptidyl-peptidase 4 (DPP-4). Glucagon(1-29) agonises glucagon receptors (GCGR) to evoke protein kinase A (PKA) activation and subsequent mobilisation of cyclic adenosine monophosphate (cAMP). Enteroendocrine L-cells of the distal gut are an open-type cell, rich in chemoreceptors and respond to digestion products of dietary carbohydrate, free fatty acids (FFA) and amino acids (AA’s) to release a number of PGDP’s into circulation **(B)**. Glicentin(1-69) is an agonist for GCGR, GLP-1R and GLP-2R, although with less affinity than their primary hormonal ligands. Additionally, glicentin may serve as a precursor to glucagon in the gut, facilitated enzymatic degradation by enzymes such as carboxypeptidases-B and -E (CP-B, CP-E). Oxyntomodulin (OXM) is a dual agonist for GCGR and GLP-1R, but shows bias towards GLP-1R. It is cleaved by DPP-4 to yield inactive OXM(3-37). Bioactive glucagon-like peptide 1 (GLP-1(7-36)) agonises target GLP-1R to evoke PKA-mediated rises in cAMP, while activation of β-arrestin is also implicated in insulin secretion. DPP-4 cleaved GLP-1(9-36) is inactive. Glucagon-like peptide 2 (GLP-2) agonises target GLP-2R to evoke rises in PKA/cAMP. It is inactivated by DPP-4 to generate GLP-2(3-23). Enzymes are indicated by yellow boxes/arrows. Receptor interactions are indicated by dashed lines, with affinity indicated by increasing thickness of the arrow. Major tissues expressing receptors are also provided.

We now understand that proglucagon is expressed in both alpha-cells of the pancreatic islets (21, 22), as well as neuroendocrine L-cells (23), primarily located in the distal ileum and colon ( (24); **Figure 1**). However, the PGDP profile is not identical in the pancreas and gut, due to differential post-translational processing of proglucagon by tissue-specific enzymes termed prohormone convertases (PC) ( (25); **Figure 1**). Broadly speaking, it is accepted that pancreatic alpha-cells mainly possess PC2, which cleaves dibasic Lys-Arg sites within proglucagon to generate glicentin-related pancreatic peptide (GRPP), glucagon, intervening peptide-1 (IP-1) and major proglucagon fragment (MPGF) ( (26, 27); **Figure 1**). In contrast, in the L-cell, proglucagon is cleaved by PC1/3 at Arg-Arg sites to yield glicentin, GRPP, oxyntomodulin (OXM), GLP-1, intervening peptide-2 (IP-2) and GLP-2 ( (23, 26); **Figure 1**). It is important to note that these distinctions are not totally sacrosanct, with a degree of crossover existing. As such, recent evidence has highlighted that the gut is a possible extrapancreatic source of glucagon ( (28); **Figure 2**), while local intra-islet GLP-1 production has also been established in alpha cells, particularly in times of beta-cell stress (29). Moreover, it is now understood that proglucagon-containing neurons are located in the solitary nucleus of the medulla oblongata (30), which utilises PC1/3 in a similar fashion to the gut to generate PGDP’s in the central nervous system (CNS) ( (31); **Figure 1**). These PGDP’s and their therapeutic exploitation will be discussed in due course.

## Glucagon

The 29 aa polypeptide hormone glucagon (**Table 1**) is the most widely recognised PGDP (9, 10), produced by PC2-mediated cleavage of proglucagon in pancreatic alpha cells ( (26, 27); **Figure 1**).

**Table 1:** Glucagon and related analogues in the management of hypoglycaemia in T1DM.

Peptide Name	Primary Sequence	Development Stage	Reference
Native glucagon	HSQGTFTSDYSKYLDSRRAQDFVQWLMNT	s.c. & i.n. formulations approved	(32–33)
NNC9204-0043	HSQGTFTSDYSKYLDSKKAQEFVQ(2xOEG-gGlu-C^18^diacid)WLLNT	Preclinical (Novo Nordisk)	(33)
Dasiglucagon	HSQGTFTSDYSKYLD-X-ARAEEFVKWLEST	Approved 2021Phase III (Zealand Pharma)	(34)

Amino acid sequences are provided in their single-letter abbreviation format. Modifications from native sequences are highlighted by red lettering. Current development stages are provided for each, as are holding companies (in brackets). “OEG-gGlu-C18 diacid” represents a fatty acid inclusion. “X” indicates an unnatural α-Aminoisobutyric acid residue.

Discovered shortly after insulin (9), glucagon and insulin are intrinsically linked, with the major metabolic actions of glucagon counteracting those of insulin (35). As such, insulin secretion from pancreatic beta-cells is stimulated largely by elevated glucose concentrations, reducing circulating glucose levels *via* inhibition of glycogenolysis and gluconeogenesis, accompanied by stimulation of glycogen synthesis in the liver (36). Furthermore, insulin stimulates glucose uptake *via* GLUT-4 translocation in adipose and muscle (37), which in turn promotes efficient metabolism of protein, lipids and carbohydrate (favouring glycolysis) (38). Conversely, hypoglycaemia following fasting, or exercise is the most potent stimulus for glucagon secretion [(39, 40); **Figure 2**].

The hyperglycaemic action of glucagon is well-established, being demonstrated as early as its discovery, with the hormone’s name reflecting this; glucagon – “the glucose agonist” (9). Hyperglycaemic actions of glucagon are mediated through promotion of glycogenolysis and gluconeogenesis in liver, whilst also inhibiting glycolysis and glycogenesis (41). Furthermore, in times of limited carbohydrate availability, glucagon promotes non-carbohydrate energy formation in the generation of lipids and ketone bodies or through the breakdown of fatty acids to acetyl-coenzyme A (42). Further research into the actions of glucagon has demonstrated a role in satiety, with acute administration in humans diminishing hunger and reducing food intake (43), whilst also stimulating energy expenditure and cardiac contractility (44, 45).

There is some debate over the receptor interactions at play in some of these biological actions, for example: given that circulating glucagon concentrations rise following a period of fasting, its involvement in food reduction seems counter-intuitive, suggesting cross-reactivity with the GLP-1 receptor (GLP-1R) (42). In the context of this article, we will consider glucagon actions mediated through agonism of its own specific G protein-coupled receptor (GPCR) the glucagon receptor (GCGR). This receptor is widely expressed, particularly in the liver, but is also found in the adrenal glands, heart, adipose tissue, GIT, and pancreas (46, 47). Binding with the receptor activates adenylyl cyclase that leads to intracellular production of cyclic adenosine monophosphate (cAMP) and subsequent activation of protein kinase A (PKA). PKA stimulates the synthesis of transcription factors including cAMP response element-binding protein (CREB) in the nucleus, a promoter of gene expression. Simultaneously, GCGR activation of phospholipase C (PLC) and subsequent increase in inositol 1,4,5-triphosphate (IP3), facilitates release of calcium ions from the endoplasmic reticulum to influence CREB-regulated transcription co-activator (CRTC2), which enhances CREB-dependent gene expression (42). Importantly, glucagon is rapidly inactivated in the circulation by enzymes, including DPP-4, to generate inactive glucagon (3–29) (48); **Figure 2**).

While considered for many years as solely a consequence of insulin deficiency, in the 1970’s the “bihormonal hypothesis”, proposed by Roger Unger, highlighted the role of an imbalance in the complex interplay between glucagon and insulin in instigating diabetic hyperglycaemia (35). Indeed, the rationale behind this longstanding hypothesis inspired research into the development of dual pump systems, sometimes termed “dual-hormone artificial pancreas”. Such pumps are regulated by a glucose sensor to deliver insulin or glucagon, as necessary, from independent pumps and are thought to be possibly more efficacious than insulin-only pumps (49), although none have successfully reached the clinic to date. We now understand that T2DM is characterised by elevated fasting glucagon levels (50), while glucose suppression following a glucose challenge is stunted (51). Furthermore, it has been suggested that postprandial hyperglucagonaemia and impaired glucagon response to hypoglycaemia are features of T1DM (52).

### Glucagon Therapeutics and Hypoglycaemia

Given glucagon’s ability to rapidly mobilise glucose from tissue stores, GCGR agonism has found valuable application in countering severe hypoglycaemia in T1DM patients, an adverse consequence of insulin therapy (53). Mild-to-moderate hypoglycaemia is defined as an event that can be self-treated, irrespective of symptom severity, or an asymptomatic blood glucose measurement of ≤3.9 mmol/L (54). It is usually managed *via* ingestion of rapidly absorbed carbohydrates, such as drinks or foods high in glucose, whereas severe hypoglycaemia requires immediate, emergency intervention (32). While intravenous (i.v.) infusion of dextrose is an option, it is now more common for patients or carers to possess an injectable glucagon preparation, which can be administered subcutaneously (s.c.) or intramuscularly (i.m.) (55). Such intervention is reliable and faster than the dextrose method, greatly reducing the risk of hypoglycaemic-induced coma and death. Rather than requiring a potentially lengthy wait for arrival of a qualified healthcare professional to perform an i.v. infusion, glucagon emergency kits simply involve reconstitution of glucagon powder, which can be injected into the patient’s leg or abdomen (32, 55). Moreover, a ready-to use autoinjector preparation termed “Zegalogue^®^” has recently gained FDA approval for management of hypoglycaemia (33), further improving ease of use. I.v. dextrose may then be required to prevent rebound hypoglycaemia (34), a potential consequence of the rapid *in vivo* inactivation of administered native glucagon (48).

Longer-acting, DPP-4 resistant analogues are in development that may address the issue of rebound hypoglycaemia. Two such analogues are the fatty-acid incorporating, NNC9204-0043 currently listed at Novo Nordisk ((34); **Table 1**), and dasiglucagon, which employs several amino acid substitutions to infer improved stability [(56); **Table 1**]. The former has only shown promise in *in vitro* settings (34), whereas dasiglucagon has very recently gained FDA approval in T1DM (56). Indeed, dasiglucagon is the active component of Zegalogue, and beyond application in prefilled injector pens, is currently in phase 3 trials as a subcutaneous infusion for treating congenital hyperinsulinaemia, and in phase 2 trials as part of a bihormonal artificial pancreas pump system alongside insulin (57). Glucagon emergency kits have been further improved with the development of intranasal (i.n.) glucagon. While not entirely novel, having been in development since the 1990’s (34), the first such product was only approved in 2019 (58). Termed Baqsimi^®^, the ready-to-use i.n. formulation has been proposed to lead to resolution of hypoglycaemia up to four times faster than injectable glucagon kits (59). The single-use preparation simply requires the user to administer one spray into either nostril, which is reported to deliver a 3 mg dose of glucagon (57).

## Glucagon-Like Peptide-1

The next PC1/3-mediated (**Figure 1**), L-cell-derived PGDP to be discussed has become a mainstay of T2DM management, representing one of the principal modern success stories of peptide therapeutic development. GLP-1 is a 29-residue (**Table 2**), gut-derived incretin hormone (77). GLP-1 is released post-prandially from L-cells [(77–79); **Figure 2**], with release influenced by the composition of each meal ingested; in particular, meals that are rich in fat and carbohydrate are known to be the primary physiological stimulus for GLP-1 secretion (78–81). Additionally, GLP-1 secretion can be triggered, not only by mixed nutrient load, but also *via* individual nutrients and bile acids. For example, oral administration of glucose alone has been shown to stimulate GLP-1 secretion in humans (82), as well as amino acids such as glutamine (83). Sodium-glucose transporter 1 (SGLT1) plays a glucose-sensing role on the L-cell surface, and although a contributor, is thought to play a lesser role than glucose transporters (GLUT) in relation to GLP-1 release (84). GLP-1 secretion is biphasic, with an early phase occurring 10-15 min after ingestion of nutrients and a second, more prolonged phase occurring 30-60 min after ingestion (81). Given the distal location of L-cells in the gut, it is unlikely that direct nutrient contact with these cells can be the sole mechanism initiating GLP-1 secretion. Thus, the autonomic nervous system, in particular the vagus nerve (which innervates a significant portion of the gut), is thought to play a role in this early phase of release, with nutrient content being more important for the second phase (85).

**Table 2:** GLP-1-based therapeutic peptides.

Peptide Name	AA Sequence	Development Stage		Reference
GLP-1(1-37)	HDEFERHAEGTFTSDVSSYLEGQAAKEFIAWLVKGRG	N/A		(60)
GLP-1(1-36)	HDEFERHAEGTFTSDVSSYLEGQAAKEFIAWLVKGR	N/A		(60)
GLP-1(7-36)	HAEGTFTSDVSSYLEGQAAKEFIAWLVKGR	N/A		(60)
N-acetyl GLP-1(7-36)	Ac-HAEGTFTSDVSSYLEGQAAKEFIAWLVKGR	Preclinical		(61)
Exendin-4 (Exenatide)	HGEGTFTSDLSKQMEEEAVRLFIEWLKNGGPSSGAPPPS	Daily - Approved 2005, Weekly- Approved 2014 (d/c 2021), Phase II-AD/PD (AstraZeneca)		(62–63)
Lixisenatide	HGEGTFTSDLSKQMEEEAVRLFIEWLKNGGPSSGAPPSKKKKKK	Approved 2016-T2DM, Phase II-AD/PD (Sanofi)		(64)
Liraglutide	HAEGTFTSDVSSYLEGQAAK*(Glu-hexadecanoyl-Glu-OH)EFIAWLVRGRG	Approved 2010-T2DM, Approved 2019-Obesity, Phase II-AD/PD, CVD (Novo Nordisk)		(65, 66)
Albiglutide	HGEGTFTSDVSSYLEGQAAKEFIAWLVKGR-{Human Albumin}	Approved 2014 (d/c 2017)-T2DM, Phase II-CVD (GlaxoSmithKline)		(67)
Dulaglutide	HGEGTFTSDVSSYLEEQAAKEFIAWLVKGGGGGGGSGGGGSGGGG{Human IgG4-Fc}	Approved 2014-T2DM, Phase II-CVD, Phase II-AD/PD (Eli Lilly)		(68)
Semaglutide	HXEGTFTSDVSSYLEGQAAK*(Glu-mPEG-17-carboxyheptadecanoyl-Glu-OH)EFIAWLVRGRG	Approved 2017- T2DM, Filed 2021-Obesity, Phase II-CVD (Novo Nordisk)		(69, 70)
Oral Semaglutide (Rybelsus)	HXEGTFTSDVSSYLEGQAAK*(Glu-mPEG-17-carboxyheptadecanoyl-Glu-OH)EFIAWLVRGRG/SNAC	Approved 2020-T2DM (Novo Nordisk)	(71–72)	
D-Ala^8^GLP-1(Lys^37^) - pentasaccharide	H(DA)EGTFTSDVSSYLEGQAAKEFIAWLVKGRK*(Pentasaccharide)	Preclinical		(73, 74)
[Gln^28^]exenatide	HGEGTFTSDLSKQMEEEAVRLFIEWLKQGGPSSGAPPPS	Preclinical		(75)
(Val^8^)GLP-1(GluPAL)	HVEGTFTSDVSSYLEGQAAKEFIAWLVK*(-Glu-PAL)GR	Preclinical		(76)

Amino acid sequences are provided in their single-letter abbreviation format. Modifications from native sequences are highlighted by red lettering. Current development stages, associated condition and holding companies (in brackets) are provided (where available) for each. FDA approval dates, and discontinuation date if applicable, are also provided where appropriate. “SNAC” represents formulation with sodium N-[8-(2-hydroxybenzoyl) amino caprylate, an absorption aid. “Ac” represents an N-terminal acetylation, “hexadecanoyl-Glu” and “carboxyheptadecanoyl-Glu” represent fatty acid attachments. “mPEG” indicates mini-polyethylene glycol addition. “PAL” indicates the addition of a palmitic acid chain. A “D” prefix before a residue indicates inclusion of the enantiomer for the naturally-occurring L form of the residue.

The biologically active forms of GLP-1 are GLP-1 (7–36)-amide and GLP-1 (7–37) which are equipotent in terms of their incretin effects [(60); **Table 2** and **Figure 2**]. However, they do not circulate equally, with GLP-1 (7–36)-amide accounting for ~80% (20, 82). Both forms of circulating GLP-1 are subject to rapid N-terminal degradation by DPP-4 (86, 87), cleaving after Ala^2^ to generate GLP-1(9-36) or (9-37) metabolites (86, 87). While GLP-1(9-36) is considered a weak antagonist of beta-cell GLP-1R (88), there is evidence suggesting that this metabolite may reduce inflammation in cardiac tissue following myocardial infarction (89). GLP-1(9-36) has also been demonstrated to promote cardiac glucose uptake similar to GLP-1(7-36)-amide (90), so the descriptor “inactive” may not be entirely accurate. Additionally, a recent study suggests that GLP-1(9-36)-amide may indirectly influence glycaemia through antagonism of GCGR on alpha-cells to influence the glucagonostatic effects of GLP-1 (91). However, the implications of any GLP-1(9-36) effects on glycaemia are thought to be relatively inconsequential in comparison to GLP-1(7-36)-amide (92).

**Figure 3:**
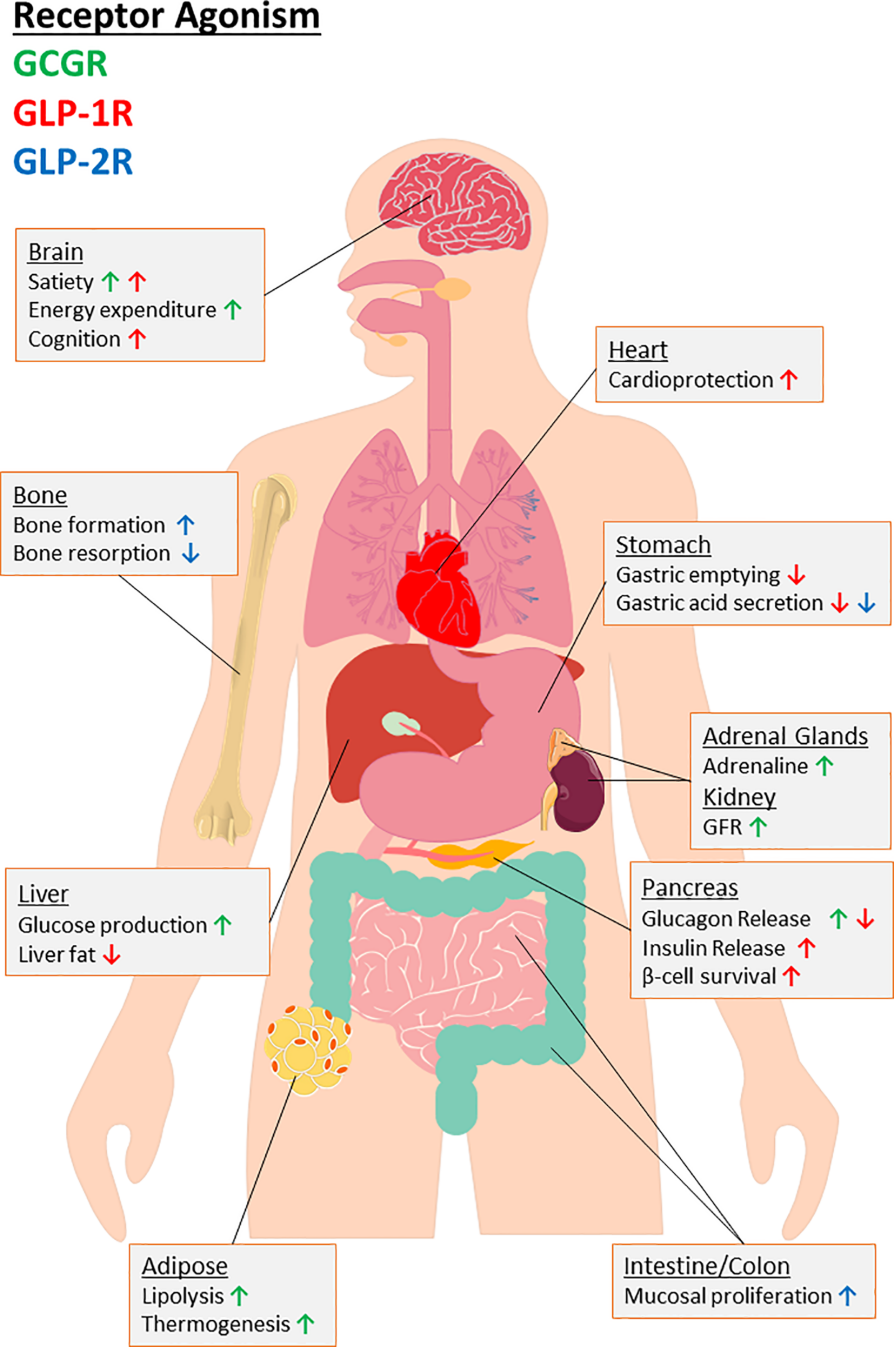
An overview of the biological consequences for agonism of target receptors of major PGDP’s, namely glucagon receptor (GCGR) and glucagon-like peptide-1 and -2 receptors (GLP-1R, GLP-2R). Organ-specific actions are provided with arrows indicating up or downregulation of specific effects to highlight the therapeutic potential for multiagonism in relation to PGDP’s. As indicated by the key, the colour of arrow indicates the receptor interactions responsible. “GFR” indicates glomerular filtration rate.

The GLP-1R is a family B, or secretin-like G-protein coupled receptor (GPCR) (93). A structurally identical GLP-1R has been identified in various tissues, for example: pancreatic tissue (alpha-, beta-, delta-cells), stomach, and intestine, as well as CNS regions including the hypothalamus and brainstem ( (81, 93); **Figure 2**). Binding of GLP-1 to its target-receptor on the beta-cell surface leads to activation of several intracellular transduction pathways (**Figure 2**). The hormone augments insulin secretion, mainly *via* stimulation of intracellular cAMP-mediated events and promotes glucose-induced biosynthesis of insulin, resulting in replenishment of insulin stores within beta-cells and reducing cell exhaustion (81, 94–96). Conversely, GLP-1 is known to suppress glucagon secretion from alpha-cells ( (97); **Figure 3**). The mechanisms behind this have been hotly debated, with it claimed to be an indirect effect mediated through increased insulin or somatostatin secretion (98, 99), while some have indicated the effect is more direct (100), especially given the presence, albeit at low expression (~10%), of GLP-1R on alpha-cells (101). Beyond this, activation of pancreas duodenum homeobox 1 (Pdx-1), a transcription factor essential for pancreatic development and beta-cell function (activated downstream from GLP-1R *via* cAMP activation), is thought to be a shared influence in these three processes (102). Prevention of beta-cell exhaustion may indirectly prevent cell death, but GLP-1 also directly influences proliferation by a number of proposed pathways including phosphatidylinositol 3-kinase (PI3-K) mediated rises in extracellular signal-related kinase (ERK) 1/2 and p38 mitogen-activated protein kinase (MAPK), as well as Pdx-1 (103). In keeping with this, exendin-4 has been shown to have no effect on proliferation or inhibition of apoptosis in beta-cell specific, Pdx-1 knockout (KO) mice (104).

Since entry into the clinic, research on GLP-1 has continued, unveiling new mechanisms behind the various benefits of GLP-1R agonists, as well as possible new applications in other conditions. With regards to diabetes, it is now well established that chronic administration of GLP-1R mimetics not only enhances insulin secretion but also positively influences overall islet function, restoring normal morphology in even severe models of diabetes (105). Additionally, the ability of exogenous GLP-1R mimetics to maintain and promote beta-cell mass through reductions in apoptosis and increases in proliferation are well established (105–108). Culture of DPP-4 resistant, N-acetyl-GLP-1 (**Table 2**) with pancreatic ductal-cells has also been shown to induce expression of genes indicative of a transition to a beta-cell like phenotype (61, 109), but translation to humans requires further study. Advances in cell-lineage tracing technology have seen the development of transgenic animal models that employ fluorescently tagged alpha- or beta-cells to identify such islet cell transitioning events in the *in vivo* setting (110, 111). Recent studies have shown that administration of liraglutide to such mice with diabetes can prevent beta- to alpha-cell transdifferentiation (112), whilst also actively driving alpha- to beta-cell conversion to help restore beta-cell mass (113–115).

GLP-1 also inhibits glucagon secretion and exerts additional extra-pancreatic actions of therapeutic value including inhibition of gastric acid secretion and gastric emptying (**Figure 3**), which help reduce post-prandial spiking of blood glucose by slowing transit of nutrients from the stomach to the small intestine (81). In addition to locally produced GLP-1 (116), GLP-1 crosses the blood-brain barrier to agonise GLP-1R within hypothalamic CNS centres, where ingestive behaviour and satiety is dictated [(117); **Figure 3**]. Increased satiety reduces food intake, with resultant weight loss being an important benefit in overweight or obese-T2DM patients. Moreover, the widespread tissue presence of GLP-1R has witnessed new physiological roles for GLP-1 beyond glycaemia and satiety such as cardioprotection ( (118, 119); **Figure 3**), enhancing bone mass and strength in preclinical models of T2DM (120), and is thought to play an important role in enhancing cognition ( (121); **Figure 3**). Additionally, a possible role for GLP-1 in resolution of hepatic steatosis (122–124) through reduction in fatty acid accumulation by activation of both macroautophagy and chaperone-mediated autophagy (125), has attracted much interest.

### GLP-1 Therapeutics and Diabetes

GLP-1 was not the first incretin hormone to be discovered, with GIP being identified almost two decades previously in 1969 (126). However, with a proposed role for GIP in development of obesity coupled with a loss of insulinotropic effect in T2DM (127), therapeutic application did not follow such a straightforward path. Thus, when a preservation of the insulinotropic effects of GLP-1 in obesity was established (128), excitement surrounding the possible therapeutic application of this newly discovered incretin hormone began to grow. Furthermore, direct comparisons of analogues of these two incretins often resulted in more favourable outcomes for GLP-1 compared to GIP (129). Nonetheless, current evidence regarding GIP-based therapy looks more promising in T2DM once glycaemic control has been re-established (130). This is perhaps evident with new compounds being developed that operate through combined activation of GLP-1R and GIPR (130), as discussed in more detail below.

Initial therapeutic investigations into GLP-1 were promising, highlighting that delivery of exogenous, native peptide had the ability to improve overall glycaemia, insulin sensitivity, beta-cell function and reduce both appetite and food intake when administered by continuous s.c. infusion over a 6 week period in patients with diabetes (131). Moreover, tachyphylaxis was not reported and the side-effect profile was favourable (131). However, due to rapid inactivation by DPP-4 (132), continuous infusion was required, making it unsuitable for regular use in a “real world” setting.

With the discovery of exendin-4, an unexpected GLP-1R mimetic isolated from the saliva of the Gila monster lizard (*Heloderma suspectum*) (62), the tide began to turn. The first 30 residues of this 39 aa peptide demonstrated 53% sequence identity with human GLP-1 (**Table 2**), but despite such variance, the peptide was proven to be a potent agonist for mammalian GLP-1R (62), effectively bringing about GLP-1R-mediated benefits on glycaemia, body weight and appetite (133). Importantly, the substitution of Ala^2^ with Gly^2^ in exendin-4 conferred resistance to DPP-4, while further sequence variations rendered the peptide less susceptible to ectopeptidases like neprilysin (NEP) (134). Studies in anaesthetised pigs has shown that GLP-1 clearance involves multiple organs including hepatic, peripheral and renal extraction, whereas exendin-4 is subject solely to glomerular filtration, which also appears to be up to two-fold slower than native GLP-1 (134). This results in an *in vivo* action of ~5 hours (63), allowing for twice daily administration as opposed to continuous infusion. Synthetic exendin-4 reached full approval for therapeutic use in humans in 2005 (Byetta™), being prescribed under the generic trade name “exenatide” and has become a highly influential and widely prescribed second- and third-line agent in T2DM, generally following failure of metformin or metformin/sulphonylurea combination (135). Indeed, oral DPP-4 inhibitors, such as sitagliptin, were approved as second-line agents in 2007 (7), while a plethora of additional GLP-1 mimetics have since gained regulatory approval for diabetes in addition to exenatide, namely the longer-acting mimetics: liraglutide, semaglutide, albiglutide and dulaglutide (**Table 2**). In contrast, attempts to discover suitable bioactive small molecule agonists of GLP-1R have failed, despite considerable efforts, due to poor potency and allosteric alteration of receptor conformation (136, 137).

### Other Potential Applications for GLP-1 Therapeutics

#### Obesity

Beyond glucose homeostasis, exciting research has highlighted extra-pancreatic benefits and new applications for established GLP-1 mimetics, many of which are exciting prospects. For example, despite the enormous upsurge in the incidence of obesity and associated complications including T2DM (138), existing drug therapies for obesity are grossly insufficient, with bariatric surgery being far more effective (139). Against this background, in 2019 liraglutide became the first GLP-1 analogue approved by the FDA, EMA and MHRA as a treatment option for obesity (65). Importantly, while glycaemic improvements undoubtedly influence weight loss, pharmacokinetic investigation in human participants suggested the effects of liraglutide on weight loss are primarily mediated through increased energy expenditure (66). Prior to regulatory approval, the “SCALE”, phase III trials demonstrated a sustained 2-year weight loss with liraglutide treatment as an adjunct to diet and exercise in non-diabetic participants (140, 141), strengthening the argument that effects are largely independent of glycaemic modulation. Additionally, 3-year follow-up demonstrated that liraglutide delayed diabetes development in patients with pre-diabetes, taking almost 3 times longer in patients receiving liraglutide (142).

Given the successful application of liraglutide in this regard and the scale of the obesity problem, other GLP-1R mimetics are beginning to be touted as treatment options for obesity. Indeed, a phase III clinical programme assessing efficacy and safety of once-weekly semaglutide (SUSTAIN) in T2DM was completed recently for s.c. semaglutide, manifesting a substantial average weight loss of 14.9% (-15.3 kg) following 68 weeks treatment (69). Additionally, a direct comparison between liraglutide and semaglutide indicated superior weight loss was attained with the latter (143). FDA approval has now been sought for semaglutide use in obesity, meaning we may be on the verge of witnessing a new treatment option available for obesity that rivals bariatric surgery.

There is also increasing interest in the therapeutic potential of combining currently available GLP-1R mimetics (**Table 2**) with other currently prescribed antidiabetic drugs. The combination of exenatide with the sodium–glucose co-transporter 2 (SGLT2) inhibitor, dapagliflozin, was investigated in the DURATION-8, phase III clinical trial which demonstrated a degree of synergy between the two agents, with improvements in short- and long-term glycaemia and weight loss exceeding either agent alone (144). Moreover, a 2-year follow-up demonstrated long-term efficacy of this combination (145). An additional phase II trial, ENERGIZE, has sought to identify the mechanism behind the apparent synergy (146), the findings of which may influence whether such a combination is advanced further.

#### Cardiovascular and Renal Benefits

The growing strength of the cardiovascular and renal benefits of established GLP-1 mimetics add another string to their bow in the management of T2DM, with cardiovascular disease (CVD) being the number one cause of death in patients with T2DM (147). As demonstrated by long-term prospective cardiovascular outcomes trials (CVOTs), which have reported over the last four years, liraglutide (LEADER), semaglutide (SUSTAIN-6), albiglutide (HARMONY OUTCOMES) and dulaglutide (REWIND) have all shown significant reductions in composite cardiovascular outcomes [(64, 119, 148); **Table 2**], indicating they may be the agents of choice when macrovascular complication risk is high in T2DM patients. These longer-acting GLP-1R mimetics elicit more favourable cardiovascular outcomes than shorter-acting agents like exenatide or its analogue lixisenatide (EXSCEL and ELIXA), which demonstrated non-inferiority, but no obvious cardiovascular benefit [(64, 119, 148); **Table 2**]. Additionally, proposed renal benefits of SGLT2 inhibitors have seen trials such as “DECLARE-TIMI 58” report reduced rates of hospitalisation due to heart failure in dapagliflozin-treated groups of T2DM patients (148). Thus, given the exploration of exenatide and dapagliflozin in the DURATION-8 and ENERGIZE trials (144–147), it may stand to reason that such a combination may be studied in relation to CVD, perhaps with a more favourable GLP-1R mimetic than exenatide. Indeed, the phase III FLOW trial is currently recruiting patients to assess the renoprotective actions of semaglutide. Thus, we await the results of this trial to determine whether semaglutide may be the GLP-1R mimetic of choice in this regard (149).

#### Cognition, Alzheimer’s, and Parkinson’s Disease

Vascular deterioration in T2DM can also be linked to cognitive impairment, with growing evidence highlighting cross-sectional and prospective associations between T2DM and cognitive impairment and diminished memory and executive function (150). Clinical studies have concluded that T2DM is a significant risk factor that can double the likelihood of developing dementia (151). It appears that a loss of insulin sensitivity in the brain (152), coupled with impaired insulin function (153), results in impaired growth factor secondary messenger cascades that are vital for cell growth, repair and synaptic function (154). GLP-1 receptor mimetics such as exendin-4 or liraglutide can reverse insulin desensitisation in the brain (155, 156). Key biomarkers for cognitive impairment such as phosphorylation of protein kinase B (AKT) and glycogen synthase kinase-3beta (GSK-3B), were reduced by liraglutide administration in diabetic rats in a time-dependent manner (153). In more practical terms, exendin-4 administration in a diet-induced obese (DIO) model reversed impaired memory formation in mice (157) and liraglutide normalised object recognition memory impairment in a similar model (158). Similar findings have been observed with DPP-4 inhibitors (159), although it is important to note that other gut hormones, particularly GIP (157), are also implicated here. Additionally, similar to CVD (145, 146), it appears that the combination of GLP-1 mimetic with SGLT-2 inhibitor may too be beneficial with regards to cognition, with DIO/STZ-mice receiving liraglutide/SGLT-2 combination therapy presenting with improved recognition and hippocampal morphology (160).

Importantly, evidence suggests that the beneficial effects of GLP-1 in relation to cognition may be independent from glycaemic improvement, with a study comparing metformin and the GLP-1 analogue (Val^8^)GLP-1(GluPAL) demonstrating that only the latter reversed memory impairment in DIO mice (76). This hypothesis is supported by the finding that GLP-1R agonists have also shown neuroprotective effects in non-diabetic patients with Alzheimer’s (AD) or Parkinson’s disease (PD) (161, 162). Long-term potentiation (LTP) of synaptic activity, the cellular correlate of memory (163), is impaired in diabetes. Liraglutide administration reversed diabetes-related LTP blockade and actively promoted LTP formation in DIO mice (157, 158), while rescuing hippocampal LTP loss in an *ob/ob* murine model of obesity-diabetes (164).

While the close relation between GLP-1 and insulin signalling is undoubtedly important in cognition, it is crucial to highlight that beyond this mechanism, GLP-1R mimetics upregulated several neuroprotective growth factors such as: insulin-like growth factor 1 (IGF-1) (165), brain-derived neurotrophic factor (BDNF) (166), glia-derived neurotrophic factor (GDNF) (164), as well as vascular endothelial growth factor (VEGF) (157, 158).

Indeed, preclinical work in rodents has illuminated both the associations between cognitive decline in AD/PD and T2DM, whilst implicating the potential of GLP-1R activation in curbing such decline (167). As such, exenatide was employed in small-scale, proof of concept, human trials in PD patients, with these trials of <100 participants indicating exenatide treatment elicited improved scores in tests of cognitive function over the course of 12 months treatment (168, 169). Moreover, a further 12 months after study conclusion, those patients receiving exenatide still achieved significantly improved cognition scores than those receiving placebo (170). With such promising results, it is unsurprising that larger scale trials were conducted, such as the phase II, ELAD trial (171), which employed liraglutide in patients with moderate AD and associated dementia. Outcomes were disappointing, with it announced in late 2020 that no difference in cerebral glucose metabolic rate or improvement in daily activity was apparent between treatment or placebo (171), although some scores of cognitive function were improved by liraglutide. Despite such disappointment, interest in GLP-1R mimetics in relation to cognitive function has not been perturbed, with a number of phase II trials recruiting in 2020 to study currently available GLP-1R mimetics in AD and PD (172). Notably, a common theme of these trials is an adjustment of treatment demographic towards patients with relatively recently diagnosed AD/PD (172).

#### Bone Fragility

Increased bone fragility is a further complication associated with diabetes, with the aetiology suspected to be due to an increase in porosity of bone, impacting on bone quality (173). Bone fragility also appears to be a feature in both T1DM and T2DM (174–176). Like cardiovascular complications, effects on bone have the potential to limit physical activity in T2DM patients. Furthermore, a role for endogenous GLP-1 in the development of diabetes-associated bone fragility has been identified, with GLP-1R KO mice presenting with reduced bone mass through increased osteoclast activity (177, 178). Given the implication of GLP-1R involvement in the aetiology of bone fragility in diabetes, research has explored the possibility of GLP-1R agonist or DPP-4 inhibitor use in the management of the condition with favourable outcomes (175, 179). Exenatide has been shown to enhance bone strength by increasing trabecular bone mass, bone formation and trabecular microarchitecture, whilst also improving collagen maturity in rodent models of diabetes (180, 181). Similarly, liraglutide significantly prevented deterioration of the quality of the bone matrix in a streptozotocin-induced, rodent model of T1DM (175). Importantly, GLP-1 is not the only incretin involved in the pathogenesis of bone fragility in diabetes, with single GIP receptor (GIPR) KO and dual GLP-1R/GIPR KO mice presenting with enhanced bone fragility (182, 183). Indeed, the unimolecular GIPR/GLP-1R/GCGR agonist, [D-Ala^2^]GIP–Oxm (**Table 4**), significantly improved bone strength and mass at both organ and tissue levels in leptin receptor-deficient, *ob/ob* obese diabetic mice (184). Possible translation of these findings from animals to humans is still required.

#### Polycystic Ovary Syndrome

There is increasing evidence in support of incretin-analogue use in polycystic ovary syndrome (PCOS) (185), an endocrine disorder which greatly impacts fertility in women, with over 10% of women of reproductive age affected by the condition (186). PCOS is a metabolic disorder that has overlap with T2DM, with patients often being overweight, and presenting with symptoms such as severe insulin resistance, hyperinsulinaemia and dyslipidaemia (187). The interrelation between PCOS and T2DM is further highlighted by the ability of bariatric surgery, specifically Roux-en-Y bariatric surgery (RYGB), to totally ameliorate both T2DM and PCOS (188, 189). Moreover, incretin function has been shown to be impaired in PCOS (187), thus application of GLP-1 mimetics in this condition is a hypothesis built on firm physiological reasoning. Although in relative infancy compared to application in T2DM, the study of application of GLP-1 mimetics in PCOS has been overwhelmingly positive (190). Liraglutide was shown to normalise irregular menstrual bleeding in PCOS patients (191), whilst improving conception rates when used at low dosage in combination with metformin (192). Indeed, it has been suggested that in obese PCOS patients with concurrent insulin resistance, GLP-1 analogues may be a better treatment option than metformin (193). Possible application of PGDPs in female fertility is worthy of further exploration.

### Innovations in Formulation and Delivery of GLP-1 Therapeutics

Since the approval of exendin-4 for T2DM, increasingly longer acting formulations of GLP-1 analogues have been developed. The first, liraglutide, a mammalian GLP-1 analogue employing conjugation to a palmitic acid chain *via* a linker coupled to the Lys^26^ residue was approved in 2010 [(194); **Table 2**]. This modification increased half-life to ~12 h, through promoting non-covalent binding to albumin and reduced renal clearance, permitting once daily administration (195). Indeed, further longer-acting analogues were developed employing several strategies. The conjugation of the native GLP-1 analogue, D-Ala^8^GLP-1(Lys^37^), to an antithrombin III (ATIII)-binding pentasaccharide, known as CarboCarrier^®^, produced a peptide with potential for once-weekly dosing [(73, 74); **Table 2**], while a once-weekly exenatide preparation (Bydureon™) which employs microspheres to form a slowly released, peptide-depot gained regulatory approval in 2014 [(196); **Table 2**]. Additionally, the once weekly preparations albiglutide and dulaglutide employ covalent interactions to attach the peptide to human albumin or a tail fragment of an IgG 4 antibody respectively, which impedes clearance (67, 68), while semaglutide achieves the same pharmacokinetic profile with non-covalent interaction with albumin (70). Such advancement has continued with a once-monthly, hydrogel preparation utilising the analogue [Gln^28^]exenatide currently undergoing development (75), while a novel osmotic minipump, termed Itca 650, is currently in phase III clinical trials (FREEDOM-1) (197). This pump administers a constant infusion of exenatide following subcutaneous implantation, reported to last for up to 12 months before requiring replacement (197).

In addition to this novel delivery method, there is growing interest in development of oral GLP-1 therapies, with preclinical data now describing bioactivity of orally delivered exendin-4 (198, 199), albeit requiring a considerably larger dose than intraperitoneal injection in mice. Most notable is a novel formulation of semaglutide that makes use of an absorption enhancer, sodium N-(8-[2-hydroxylbenzoyl] amino) caprylate (SNAC), designed to protect peptides from proteolytic degradation and promote absorption across the gastric mucosa [(71); **Table 2**]. Phase II trials comparing oral to s.c. semaglutide in diabetes management revealed comparable improvements in glycaemia when compared to placebo, but notably oral treatment attained slightly greater weight loss over the 26 week study (-6.9 kg/-7.6%, compared to -6.4 kg/-7.2%) (71). This therapeutic has recently gained FDA approval following successful phase III trials (PIONEER-7) in T2DM patients and provides significantly better improvements in glycated haemoglobin (HbA_1c_) than sitagliptin in T2DM (200). Like previously available oral antidiabetics (7), oral semaglutide is taken once-daily as a tablet formulation, being prescribed under the brand name Rybelsus^®^ (201). Moreover, as part of the PIONEER trial program, oral semaglutide was studied in patients with renal impairment and demonstrated favourable outcomes (202), possibly indicating that like s.c. semaglutide there was cardiovascular benefit (118). However, when outcomes were assessed upon completion of PIONEER-6 non-inferiority compared to placebo was evident (72), but there was no obvious cardiovascular benefit. These new findings are highly relevant and should lead to greater patient acceptability and compliance in treatment of T2DM and other disorders, as compared to traditional injection route for peptide therapies.

## Glucagon-Like Peptide-2

The discovery of GLP-1 and GLP-2 occurred simultaneously following the cloning of cDNAs and genes encoding mammalian proglucagon in the early 1980s, with experiments unveiling the sequences of two novel glucagon-like peptides (15, 16). At that time, the biological functions had not been described for either hormone, with the insulinotropic actions of GLP-1 reported in 1987 (96). This delay was due to the lack of bioactivity of GLP-1 (1–37) (203), which hampered progress until the truncated peptide GLP-1 (7–36)-amide was uncovered (204). Perhaps, as a result of subsequent research focusing on the exciting prospect of exploiting GLP-1 as a potential antidiabetic agent, GLP-2 based research may be considered somewhat less intense, with the biological action as a growth promoter in gut not being uncovered until almost a decade after actions of GLP-1 (205).

The development of the first GLP-1/GLP-2 secreting GLUTag cell-line represents a starting point in the elucidation of the biological function of GLP-2. This cell line was produced *via* the creation of a transgenic mouse model with GLP-1/2 secreting tumours in the colon, from which L-cells could be extracted and immortalised (206). An observation was made that these animals all exhibited marked enlargement of the small bowel following tumour-induction, inspiring the hypothesis that a PGDP secreted by these tumours must have been responsible for the intestinotrophic activity (205). Interestingly, Bloom had reported the first enteroglucagonoma patient with small intestinal villous hypertrophy, malabsorption, as well as colonic and jejunal stasis some 20 years earlier (207). However, the question remained as to which hormone, or hormones, were responsible. Initially, the intermediary peptide, glicentin, was identified to elicit intestinotrophic action (208). However, subsequent administration of synthetic GLP-2 into mice indicated that GLP-2-mediated increases in small bowel weight surpassed those seen with glicentin (209), making it the more likely instigator.

As their name suggests, both GLP hormones are closely related with both being synthesised by the action of PC1/3 and secreted from intestinal L-cells of the distal gut (**Figure 1**) (25, 210). Following liberation from proglucagon, the 33 residue GLP-2 is secreted post-prandially in a biphasic fashion from nutrient-sensing L-cells (**Figure 2**), particularly in response to carbohydrates and lipids contained within luminal contents [(211); **Figure 2**]. Notably, the distal location of these cells indicates a neural pathway must be involved, given plasma GLP-2 levels (along with other L-cell-derived hormones) are shown to rise rapidly following ingestion (212).

GLP-2 exerts its actions through agonism of its own target receptor, a GPCR termed the GLP-2 receptor (GLP-2R) [(25); **Figure 2**]. The receptor is widely expressed throughout the entirety of the gut and is highly specific for GLP-2, with other PGDPs demonstrating relatively low affinity (213). Similar to GLP-1, agonism of the GLP-2R evokes a rise in intracellular cAMP and subsequent PKA activation, however, intracellular calcium remains unchanged [(214); **Figure 2**]. Activation of the receptor directly reduces enterocyte apoptosis and increases crypt cell proliferation, which operates in tandem to increase microvilli height [(215); **Figure 3**]. The hormone has also been demonstrated to improve intestinal blood flow, decrease gut motility and inhibit gastric acid secretion [(216); **Figure 3**]. There is some evidence that GLP-2 is produced in small functional amounts within pancreatic islets, but the alternative processing of proglucagon by PC1/3 in alpha-cells to give GLP-1 under conditions of cellular stress is likely much more significant (217).

### GLP-2 Therapeutics and Short Bowel Syndrome

The intestinotrophic properties of GLP-2 were an attractive prospect in development of therapeutics for conditions such as short-bowel syndrome (SBS), usually a consequence of surgical removal of a section of the bowel in Crohn’s disease (218). This condition is characterised by malabsorption as a result of chronic diarrhoea with further dehydration and weight loss, and depending on severity, the overall quality of life can be greatly impaired. The condition can be managed by parenteral nutrition (PN) and hydration, however, in the long-term this increases the likelihood of infection and potentially sepsis (219). Additionally, patients have a strict reliance on PN which can impede mobility, further impacting on quality of life. Hence, a medication with the ability to manage the condition and reduce the need for PN was highly sought after.

In support of GLP-2 use in SBS, endogenous levels have been shown to rise following excision of bowel (220), while preclinical data showed promising improvements in bowel mass in rats receiving GLP-2 infusion following 75% removal of the mid jejuno-ileum (221). Moreover, infusion of GLP-2 in patients in whom the terminal ileum and colon had been resected, improved intestinal absorption and nutritional status (222). Thus, GLP-2R has clear application in treatment of the condition. As is the case with GLP-1(7–36), GLP-2 is rendered inactive by enzymatic N-terminal dipeptide (His^1^-Ala^2^) removal by DPP-4, producing the major fragment GLP-2 (3–33) (205). Thus, in order to be therapeutically viable, the native hormone must be modified to facilitate exogenous administration.

Substitution of the penultimate Ala^2^ for Gly^2^ (as found in exendin-4) enabled the development of [Gly^2^]GLP-2 (**Table 3**), a DPP-4 resistant, long-acting GLP-2 mimetic (214). The peptide employed single amino acid substitution and presented a more specific approach than blanket DPP-4 inhibition (222). The analogue was later named “teduglutide” and demonstrated early promise in a dose-range pilot study in human SBS patients (227). Subsequent phase III clinical trials confirmed beneficial effects in several cohorts of SBS patients, manifesting in improved intestinal morphology, renal function as well as a favourable side-effect profile (223, 228). Furthermore, treatment reduced reliance on PN in many patients (223), while a portion of previously dependent patients was able to completely discontinue PN (229). Teduglutide was subsequently approved by the FDA in 2012 and is prescribed under the trade names Gattex^®^ in the USA and Revestive^®^ in Europe (**Table 3**).

**Table 3:** GLP-2-based therapeutic peptides.

Peptide Name	AA Sequence	Development Stage	Reference
Native GLP-2(1-33)	HADGSFSDEMNTILDNLAARDFINWLIQTKITD	N/A	(205)
[Gly^2^]GLP-2	HGDGSFSDEMNTILDNLAARDFINWLIQTKITD	Preclinical	(209, 214)
Teduglutide	HGDGSFSDEMNTILDNLAARDFINWLIQTKITD	Approved 2012-SBS (Shire-NPS Pharmaceuticals)	(222–223)
Apraglutide	HGDGSFSDE-Nle-(DF)TILDLLAARDFINWLIQTKITD	Phase III-SBS (VectivBio)	(224, 225)
Glepaglutide	HGEGTFSSELATILDALAARDFIAWLIATKITDKKKKKK	Phase III-SBS (Zealand Pharma)	(226)

Amino acid sequences are provided in their single-letter abbreviation format. Modifications from native sequences are highlighted by red lettering. Current development stages, and associated condition, and holding companies (in brackets) are provided (where available) for each are provided for each. A “D” prefix before a residue indicates inclusion of the enantiomer for the naturally-occurring L form of the residue. “Nle” indicates the addition of a norleucine residue.

Following the success of teduglutide, further GLP-2 analogues are currently in development, with research aimed to improve the ~5 h circulating half-life of teduglutide (230). Apraglutide ([Gly^2^, Nle^10^, D-Phe^11^, Leu^16^]-GLP-2) employed further substitutions (**Table 3**), identified through structure-activity relationship studies of lipophilic amino acid substitutions in positions 11 and 16 of teduglutide, and has been shown to prolong *in vivo* bioactivity through reduced renal clearance in rodents (224). Similar findings were observed in monkey and mini-pig (225), whilst exhibiting excellent specificity and potency for the GLP-2R. The peptide was more efficacious than both teduglutide and another GLP-2 analogue in development, glepaglutide [(226); **Table 3**], and has started recruiting for phase III clinical trials in SBS patients (231). That said, glepaglutide has a reported half-life of 50 h and has also entered phase III clinical trials (226). It employs nine amino acid substitutions and a C-terminal tail of six Lys residues (**Table 3**). The analogue forms a subcutaneous depot at the injection site, from which glepaglutide and its active metabolites are gradually released into the circulation. Phase II trials indicated the analogue was well absorbed, effective and tolerated (226). Thus, apraglutide and glepaglutide may represent an exciting new step in development of GLP-2 analogues, emulating the success of long-acting GLP-1 analogues, which can be administered at less frequent intervals than currently available once-daily preparation, teduglutide.

### GLP-2 Therapeutics and Osteoporosis

An additional similarity to GLP-1 research is the pursuit of new therapeutic applications. With the widespread expression of GLP-2R (213), it was postulated that GLP-2 may have application in the management of osteoporosis. Osteoporosis is a condition characterised by bone mass reduction and microarchitecture impairment caused by an imbalance in bone formation and resorption, increasing the risk of fractures (232). Moreover, the prevalence of osteoporosis continues to surge in accordance with an increasingly ageing population (233). A number of the widely-prescribed, anti-resorptive drugs, particularly bisphosphonates, are believed to possess unfavourable side-effect profiles (234), thus alternative treatment options are being sought. Indeed, the involvement of gut hormones in bone mass and formation has been widely researched, with the roles of GLP-1, as well as GIP (175), discussed above.

However, unlike these related gut hormones, the role and indeed application of GLP-2 with respect to bone mass is more divisive. In initial studies of GLP-2 in SBS, an additional observation was made that, following 5 weeks treatment, patients presented with significantly increased spinal areal bone mineral density (222). Subsequently, it was demonstrated that s.c. GLP-2 administration reduced bone resorption in post-menopausal women while not affecting bone formation (235). However, the findings in SBS patients were refuted, with a later study reporting that an intact bowel is required for exogenous GLP-2 administration to have such an effect (236). Additionally, unlike GIPR, an equivalent GLP-2R has not been identified on human osteoclasts (237), indicating that its actions are indirect, with inhibition of parathyroid hormone (PTH), mediated by activation of GLP-2R on PTH gland, suggested to be the mediator of its effects on bone resorption (236). Moreover, a small-scale trial in healthy males employed GIPR antagonists to confirm the antiresorptive effects of GLP-2 are independent of this receptor (238). The mechanisms behind the bone actions of GLP-2 require further investigation to firmly establish a link.

Despite this, several studies support the involvement and potential use of GLP-2 in bone formation in some capacity. In a study of postmenopausal women with concurrent T2DM, it was revealed that ingestion of a mixed nutrient meal saw a reduction in biomarkers for bone fragility, coupled with a rise in GLP-2 levels (239), indicating the importance of the gut. However, this study did not ascertain the involvement of other gut hormones. These findings are supported by more recent work in ovariectomised rats, an animal model replicating postmenopausal osteoporosis. It was established that 4 weeks s.c. administration of GLP-2 resulted in improvements of bone architecture and mass through both promotion of bone formation and a reduction in resorption (240). Interestingly, studies of GLP-2 effects on bone have all employed human GLP-2, as opposed to longer-acting analogues. Furthermore, i.v. administration of a high dose of GLP-2 was outperformed by lower doses of s.c. GLP-2 in terms of reducing bone resorption (241). Thus, given longer acting, s.c. teduglutide is currently available, as well as other enzyme resistant analogues in development, their potential use for therapy of osteoporosis is exciting. Moreover, given the involvement of several gut hormones in this gut-bone axis (242), coupled with the success of unimolecular multiagonists with relation to bone improvements (184), it stands to reason that incorporation of a GLP-2R agonising component may improve the efficacy of such agents in promoting bone density.

## Other Potential Proglucagon-Derived Therapeutics

### Oxyntomodulin

Oxyntomodulin (OXM) was discovered as a fragment of glicentin (243, 244), sharing substantial sequence homology and essentially the entire 29 amino acid glucagon molecule with an additional C-terminal octapeptide, IP-1, resulting in 37 residue OXM ( (245, 246); **Figure 1** and **Table 4**).

**Table 4:** Oxyntomodulin-based therapeutic peptides.

Peptide Name	AA Sequence	Development Stage	Reference
Native OXM	HSQGTFTSDYSKYLDSRRAQDFVQWLMNTKRNKNNIA	N/A	(245)
(D-Ser^2^)Oxm[mPEG-PAL]	H(DS)QGTFTSDYSKYLDSRRAQDFVQWLMNTKRNKNNIA-[mPEG-PAL]	Preclinical	(247)
Dogfish OXM	HSEGTFTSDYSKYMDNRRAKDFVQWLMSTKRNG	Preclinical	(248)
Ratfish OXM	HTDGIFSSDYSKYLDNRRTKDFVQWLLSTKRNGANT	Preclinical	(248)
[D-Ala^2^]GIP–Oxm	YDAEGTFISDYSKYLDSRRAQDFVQWLMNTKRNRNNIA	Preclinical	(184)
OX-SR	Structure N/A	Preclinical	(249)
LY3305677	Structure N/A	Phase II-T2DM/Obesity (Eli Lilly)	(250, 251)
DualAG	HSQGTFTSDYSKYLDSRRAQDFVQWLMNTKRNKNNIA-Chol	Preclinical	(252)
GLPAG	HSEGTFTSDYSKYLDSRRAQDFVQWLMNTKRNKNNIA-Chol	Preclinical	(253)

Amino acid sequences are provided in their single-letter abbreviation format. Modifications from native sequences are highlighted by red lettering. Current development stages, associated condition and holding companies (in brackets, where available) are provided for each. A “D” prefix before a residue indicates inclusion of the enantiomer for the naturally-occurring L form of the residue. “mPEG” indicates mini-polyethylene glycol addition. “PAL” indicates the addition of a palmitic fatty acid chain. “Chol” represents attachment of a human cholesterol fragment. “Structure N/A” represents a molecule for which the amino acid sequence has not been disclosed by authors.

Like other gut-based PGDPs, OXM is released post-prandially from L-cells (254). OXM increases energy expenditure and physical activity, promotes weight loss and improves glycaemia in humans (254, 255). No specific OXM receptor is known to exist; rather, the peptide acts as a dual agonist for GCGR and GLP-1R (**Figure 2**), although it binds to both with lower affinity than either of their primary ligands (256, 257). In the current thinking, OXM-mediated weight loss is believed to be elicited through activation of the GCGR, bringing about anorectic actions and increased energy expenditure [(258); **Figure 3**]. In contrast, GLP-1R agonism accounts for improved glucose homeostasis through augmented insulin secretion, overcoming the hyperglycaemic actions of GCGR activation [(259); **Figure 3**]. Mechanistic studies reveal that OXM behaves as a differential agonist depending on the receptor, acting as a full agonist in recruiting β-arrestin 2 to the GCGR, but partial agonist in recruiting β-arrestin 1 and 2 and GPCR kinase 2 to the GLP-1R (260). Furthermore, some data suggests that OXM is a GLP-1R-biased agonist relative to GCGR (260).

### Oxyntomodulin Therapeutics and Obesity/Diabetes

As alluded to above, the ability of OXM to effectively activate both GCGR and GLP-1R, thereby improving blood glucose and body weight, is attractive for the development of peptide therapeutics for obesity/T2DM, provided an appropriate receptor balance is struck. Like all other PGDPs, OXM is subject to rapid inactivation by DPP-4 which targets cleavage after the N-terminal Ser^2^ residue (261). This rapid inactivation precludes use of the unmodified hormone as a therapeutic. Thus, while initial studies demonstrated that native OXM decreases food intake and enhances energy expenditure in both healthy and obese human volunteers, these employed undesirably frequent dosing of three- or four-times daily (262, 263).

As with GLP-1, DPP-4 resistant forms of OXM are required therapeutically and given the sequence similarities between the two peptides, successful approaches taken with GLP-1 can be applied to OXM (261, 262). One example, (D-Ser^2^)Oxm[mPEG-PAL] (**Table 4**), employed substitution of the naturally occurring L-Ser^2^ with the enantiomer D-Ser^2^ to promote DPP-4 resistance, while further utilising C-16 palmitic acid conjugation *via* a mini-PEG linker at the C-terminus to reduce renal clearance and improve circulating half-life (247). The resulting peptide was fully resistant to DPP-4, whilst clearly retaining bioactivity: increasing cAMP in both GLP-1R and GCGR transfected cell lines, as well as enhancing insulin release from clonal pancreatic beta-cells (247). Additionally, daily administration of (D-Ser^2^)Oxm[mPEG-PAL] to *ob/ob* mice decreased food intake and body weight, whilst increasing plasma and pancreatic insulin and improving glucose tolerance (247). Several biomarkers of obesity were also improved, including increased adiponectin with reductions in both visfatin and triglyceride concentrations (247). The OXM analogue also exerted beneficial effects on blood glucose control in STZ-diabetic mice, including elevations in total islet and beta-cell areas associated with an increase in the number of smaller-sized islets and enhanced islet proliferation (264). A follow-up study with (D-Ser^2^)Oxm[mPEG-PAL] in transgenic mice with fluorescently tagged alpha cells also demonstrated highly favourable effects on islet cell transdifferentiation (265). Interestingly, another study employed dogfish and ratfish oxyntomodulin peptides (**Table 4**), which despite numerous sequence variations from human OXM, remained effective at mammalian GCGR and GLP-1R (248). This suggests a possible early advantage of such dual receptor actions in evolutionary terms.

The therapeutic applicability of enzymatically stable OXM analogues is clear and a number of analogues are in various stages of development for potential use in T2DM therapy (**Table 4**). However, it has been demonstrated that a balance in GCGR/GLP-1R agonism must be reached when designing OXM analogues, with a number of examples demonstrated to induce hyperphagia (266). OXM analogues with Glu^3^ substitution favour GLP-1R activation and do not exhibit an orexigenic effect (266), hence, it is assumed that such an effect must be mediated *via* GCGR agonism (266, 267). However, with the development of OX-SR, a sustained-release oxyntomodulin analogue which employs 5 central, depot-forming, amino acid substitutions between residues 16-27 of the human peptide (exact sequence not disclosed by authors), an OXM analogue capable of bringing about GCGR-mediated increases in energy expenditure was developed, and despite having an orexigenic effect actually elicited 2% weight loss following 3 days administration in rats (249). In this respect, while OX-SR was proven to agonise both receptors *in vitro*, the analogue showed greater affinity for GCGR than GLP-1R (249). More prolonged studies, including those in models of diabetes are required to investigate the long-term consequences of such prolonged exposure to GCGR and GLP-1R activation by OX-SR, but the peptide does represent a potential once-weekly OXM formulation (249). Excitingly, regulatory approval of the first OXM analogue may be on the horizon, with the long acting, fatty-acid derivatised analogue LY3305677 (sometimes termed IBI362) currently in separate phase II clinical trials investigating management of T2DM and obesity (250, 251).

### Glicentin and Glicentin-Related Pancreatic Peptide

Glicentin is a product of PC1/3 proglucagon processing, while GRPP glicentin-related pancreatic peptide (GRPP) is a product of PC2 processing in the pancreas [(22, 25); **Figure 1**]. Radioimmunoassay of gut extracts revealed substances with glucagon-like immunoreactivity that cross-reacted with antibodies directed towards the N-terminus of glucagon (268), with further investigation identifying two related proteins, one appearing to be a fragment of the other. Firstly, the 69 residue, N-terminal proglucagon fragment glicentin (243), which contained the entire glucagon sequence attached to an N-terminal portion later identified as GRPP (269, 270). The smaller fragment was essentially glucagon attached to a C-terminal octapeptide called intervening peptide-1 (IP-1) (271), later this C-terminally extended glucagon was denominated as oxyntomodulin.

We now know glicentin is released post-prandially from L-cells of the distal ileum and colon, particularly in response to glucose, lipids and amino acids, especially arginine, entering the duodenum [(272–274); **Figure 2**]. The hormone elicits a number of physiological effects such as a paracrine role in promoting intestinal growth and regulating motility (275), as well as playing a role in glucose homeostasis through augmenting insulin secretion and inhibiting glucagon secretion (276). However, no glicentin receptor has yet been identified, but the hormone has been shown to agonise and elicit cAMP production following binding to glucagon, GLP-1 and GLP-2 receptors [(277, 278); **Figure 2**]. Additionally, earlier work with glicentin suggested that its actions were largely dependent upon the degradation of the hormone into smaller molecular fragments (279), possibly including carboxylase-mediated generation of glucagon (**Figure 2**). This may, in part, explain why there has been relatively little research exploring development of glicentin-based therapeutics. Furthermore, it is likely that a lack of commercialised detection methods for glicentin have hindered its overall investigation and therapeutic application (280). However, with increasing availability and affordability of capable assays and given the increasing interest in peptide therapeutics, we may see renewed interest in this PGDP (281). Moreover, it has recently been put forward that post-surgery rises in glicentin, along with OXM, are the best predictors of decreased in intake of energy-dense foods and weight loss following RYGB, more so than even GLP-1 (282). Whether this translates to functional involvement remains unclear, and instead it is postulated that increased glicentin levels are a useful indicator of improved overall L-cell function (282).

As discussed above, GRPP is one of the products of PC2 processing of proglucagon in pancreatic alpha-cells (25, 26). A 30 residue, N-terminal fragment of proglucagon (**Figure 1**), GRPP was discovered after glicentin using glicentin-specific antibodies in pancreatic extracts (269). Structural elucidation highlighted that the peptide was identical to the N-terminus of gut-derived glicentin, hence the name glicentin-related pancreatic peptide (269). Despite its discovery almost four decades ago, research on this PGDP is sparse, but earlier experiments in dogs suggest the peptide may influence glucose homeostasis through increasing plasma insulin and decreasing plasma glucagon (283). A more recent study utilised isolated-perfused pancreas and liver from rats to pursue a detailed investigation of the physiology of this peptide (284). In contradiction of initial findings, this study demonstrated that while glucose output from the liver remained unaffected, GRPP brought about potent inhibition of glucose‐stimulated insulin secretion in perfused pancreas, with cAMP assay indicating that these actions were not mediated through either GLP-1R or GCGR, meaning an unidentified receptor may be at play (284). Given the lack of physiological data surrounding GRPP, it is unsurprising that no therapeutic exploration has been made on this PGDP.

## Multiagonists

Unimolecular multiagonists represent an exciting future in the therapeutic application of PGDPs, with increasingly complex and experimental molecules being developed. As briefly mentioned above, RYGB surgery induces rapid remission of T2DM in 70-80% patients (285). Importantly, secretion and action of a number of gut hormones, including the PGDPs GLP-1, GLP-2, OXM and glicentin, together with PYY, GIP, cholecystokinin (CCK), neurotensin (NT) and secretin, are positively modulated in concert following RYGB (286). These are thought to be major determinants in the improvements of appetite, body weight, glucose tolerance and insulin sensitivity demonstrated post-surgery (286). Thus, given high costs, limited availability and potential risks associated with surgical procedures, there is a current focus on designing multiagonist molecules with the ability to emulate the post-surgical, hormonal mechanisms of RYGB, which have the potential to be more widely available to patients than surgery. Additionally, they have the potential to evoke an array of positive actions within various organs (**Figure 3**), and such molecules could surpass advantages observed with individual peptides.

Earlier research employing combinations of single gut hormones or analogues provided a sound basis for the application of multi-agonism in T2DM (287). Indeed, with the combination of liraglutide plus an acylated GIP analogue (288), synergy was demonstrated leading to improved glucose-lowering and insulinotropic actions in obese-diabetic mice compared to either of the individual incretin analogues alone. Furthermore, recent combination studies have further strengthened the idea that combined exogenous peptide administration can effectively emulate the benefits of RYGB. As such, infusion of a multi-peptide preparation of GLP-1, OXM and PYY (3–31, 35–39) termed “GOP”, can replicate the postprandial levels of these hormones observed after RYGB, and can safely bring about 32% reduction in food intake in a standardised meal test (289). Moreover, continuous GOP infusion, delivered by pump over a 4-week period in obese patients with prediabetes or diabetes, resulted in improvements in glucose tolerance which surpassed those of RYGB (290).

To date, a number of unimolecular double- and triple-agonists have been developed with several being actively pursued for clinical application (**Table 5**). The majority of these typically employing a GLP-1R agonist component combined with another gut hormone, often an incretin or other PGDP.

**Table 5:** Multiagonists based on proglucagon-derived peptides in development.

Peptide Name	AA Sequence	Target Receptors	Development Stage	Reference
Dual Agonists				
Cotadutide	HSQGTFTSDK-(Palmitoyl-E)SEYLDSERARDFVAWLEAGG	GLP-1R/GCGR	Phase II-T2DM, NASH/NAFLD (AstraZeneca)	(291–292, 293)
Efinopegdutide	Structure N/A	GLP-1R/GCGR	Phase II-NASH/NAFLD (Merck & Co)	(294–295)
Tirzepatide	Y-Aib-EGTFTSDYSI-Aib-LDKIAQK*(C20 diacid γ-E)AFVQWLIAGGPSSGAPPPS	GLP-1R/GIPR	Phase III-T2DM, Phase II-NASH (Eli Lilly)	(296–297)
NN9389	Structure N/A (GIP/Semaglutide Preparation)	GLP-1R/GIPR	Phase I-T2DM (Novo Nordisk)	(298)
CT-868	Structure N/A	GLP-1R/GIPR	Phase I-T2DM (Carmot Therapeutics)	(298)
TAK-094	Structure N/A	GLP-1R/GIPR	Phase I-T2DM (Takeda Pharmaceuticals)	(298)
(pGlu-Gln)-CCK-8/exendin-4	pEQDY-(SO_3_H)-MGWMDF-(AEEAc-AEEAc)-HGEGTFTSDLSKQMEEEAVRLFIEWLKN	GLP-1R/CCK1R	Preclinical	(299)
C2816	HGEGTFTSDLSKQMEEEAVRLFIEWLKN-[PEG^4^]-Nle-GWK(Tac)D-NmeF	GLP-1R/CCK1R	Preclinical (MedImmune/Astrazeneca)	(300)
GUB06-046	HXEGTFTSDLSRLLEGAALQRFIQWLV	GLP-1R/SCTR	Preclinical (Gubra)	(301)
EP45	HGEGTFTSDLSKQMEEEAVRLFIEWLKNGGPSSRHYLNLVTRQRY	GLP-1R/NPY2R	Preclinical	(302)
Exendin‐4/xenin‐8‐Gln	HGEGTFTSDLSKQMEEEAVRLFIEWLKN‐(AEEAc‐AEEAc)‐HPQQPWIL	GLP-1/NTSR1	Preclinical	(303)
Triple Agonists				
YAG-glucagon	Y[DA]QGTFTSDYSIYLDSNVAQDFVQWLIGG	GLP-1/GIPR/GCGR	Preclinical	(304)
Exendin‐4/gastrin/xenin‐8‐Gln	HGEGTFTSDLSKQMEEEAVRLFIEWLKN‐(AEEAc‐AEEAc)‐YGWLDF ‐(AEEAc‐AEEAc)‐HPQQPWIL	GLP-1/CCK2R/NTSR1	Preclinical	(305)
Exendin‐4(Lys^27^γ‐Glu‐PAL)/gastrin/xenin‐8‐Gln	HGEGTFTSDLSKQMEEEAVRLFIEWLK(γ‐E‐PAL)N‐(AEEAc‐AEEAc)‐YGWLDF ‐(AEEAc‐AEEAc)‐HPQQPWIL	GLP-1/CCK2R/NTSR1	Preclinical	(306)
LY3437943	Structure N/A	GLP-1/GIPR/GCGR	Phase I (Eli Lilly)	(269)
HM15211	Structure N/A	GLP-1/GIPR/GCGR	Phase II (Hanmi Pharmaceuticals)	(307–308)
TA	HXQGTFTSDK*(γE-C16)SKYLDERAAQDFVQWLLDGGPSSGAPPPS	GLP-1/GIPR/GCGR	Preclinical	(309, 310)

Amino acid sequences are provided in their single-letter abbreviation format. The receptor targets for each molecule, as well as current stage of development and holding companies (in brackets, where available) are provided for each. A “D” prefix before a residue indicates inclusion of the enantiomer for the naturally-occurring L form of the residue. “PAL” indicates the addition of a palmitic fatty acid chain, “PEG” indicates a polyethylene glycol linker. “Aib”, “Nle” and “NmeF” indicate the addition of an unnatural 2-aminoisobutyric acid, norleucine or N-methyl phenylalanine residues. “pE” indicates pyroglutamine. “K(Tac)” indicates inclusion of a side-chain substituted (o-tolyamino)carbonyl lysine residue. “(AEEAc‐AEEAc)” indicates a commonly employed linker molecule between peptide regions. “γE-PAL” represents a fatty acid attachment.

### Dual Agonism With GLP-1 and Glucagon

As previously discussed, the notion of GCGR agonism in pursuit of a therapeutic for T2DM, or its related conditions, seems counterintuitive. However, given the surprising beneficial effects of OXM agonists in T2DM, the benefits of targeting these two receptors in tandem was clearly demonstrated (**Figure 3**). Additionally, the structural similarity between the two PGDPs was clearly demonstrated (**Table 4**). As such, this combination pioneered unimolecular PGDP-based research, with the cholesterol-conjugated OXM analogue DualAG and the glucagon, GLP-1 chimeric peptide “Aib^2^ C^24^ chimera 2 lactam 40K” both showing preclinical promise in murine, DIO models of obesity-diabetes [(252, 311); **Table 4**].

While a number of GLP-1/glucagon based peptides have been generated (**Table 5**), many have witnessed therapeutic pursuit abandoned. Currently, a molecule of particular promise is cotadutide (formerly MEDI0382). Cotadutide is a linear, chimeric peptide employing important residues from both glucagon and GLP-1 into its sequence (**Table 5**), with a palmitoyl FA attachment on Lys^10^ to prolong circulating half-life (253). The peptide is reported to be a balanced dual-agonist for GLP-1 and GCGR, which brought about significant weight loss through improved glycaemia in DIO mice and non-human primates, being more effective than liraglutide alone (253). The concept of balance in respect to such molecules is crucial, as it is important to maximise weight loss whilst minimising the potential to cause hyperglycaemia, with as little as 10% relative GLP-1 sequence contribution minimising hyperglycaemia whilst retaining weight loss (312). The effects on glycaemia were supported by acute administration studies in humans, however a slower dose titration was deemed necessary to avoid adverse effects on gastric emptying (291).

When assessed in phase II trials in T2DM patients, slower titration of cotadutide was employed to reflect such findings (313). This study revealed that daily administration in patients with controlled T2DM improved overall glycaemic control, as measured by HbA_1c_, which was associated with sustained weight loss following 41 days daily administration (313). Subsequently, it was revealed that these positive effects on glycaemia were likely the result of improved gastric emptying and postprandial insulin response (292). Additionally, patients presented with significant improvements in liver fat, with levels falling by 39% (313), which was notable given an equivalent fall in levels with liraglutide takes around 6 months (314). These findings on liver fat have seen a refocus of research toward application in non-alcoholic fatty liver disease (NAFLD) and steatohepatitis (NASH) (293), both common consequences of uncontrolled T2DM (315). The study revealed that cotadutide’s actions on the liver to reduce lipid content, drive glycogen flux and improve mitochondrial turnover and function are directly mediated through modulation of hepatic GCGRs, while metabolic improvements mediated *via* agonism of extrahepatic GLP-1Rs further enhanced improvement (293). A similar story is unfolding for the GLP-1/GCGR agonist efinopegdutide (formerly HM12525A), a longer acting agonist which employs modified exendin-4 conjugated to human IgG, facilitating once-weekly administration ( (294); **Table 5**). The peptide appealed as a treatment for T2DM due to promising preclinical results which demonstrated lipolytic and insulinotropic effects in diabetic mice (316). However, potent lowering effects on cholesterol and liver fat have seen this analogue also repurposed as a potential NAFLD/NASH medication (295).

### Dual Agonism With GLP-1 and GIP

With synergy demonstrated by administration of liraglutide plus an acylated, enzyme resistant GIP analogue (288), the value of developing molecules targeting these two incretin receptors was evident. As such, a number of unimolecular GLP-1/GIP agonists have been developed and are at various stages of clinical testing (**Table 5**).

One particular success story involves a molecule termed tirzepatide (formerly LY3298176) (296). The peptide is a linear, 39 aa peptide containing two unnatural residues and a C20 diacid fatty acid attached *via* a linker to Lys^20^ (**Table 5**), all of which contribute to a circulating half-life of ~5 days, which permits once weekly dosing (296). Tirzepatide may be considered a GIP-based analogue, sharing greater sequence homology with GIP than GLP-1 (particularly at the N-terminus) (**Table 5**), with GLP-1R agonism induced *via* aa substitution (296, 317). The peptide was shown to effectively lower blood glucose *via* insulinotropic actions at both receptors in preclinical studies in mice, while phase I trials revealed effective weight loss in T2DM patients and good tolerability (296). Interestingly, *in vitro* mechanistic studies suggest the peptide is biased towards the GIPR, activating with equipotency to native GIP whilst having 5-fold weaker affinity than native GLP-1 at GLP-1R, with a preference to initiate cAMP mobilisation to enhance insulin secretion (317), which may be of particular benefit in obesity-diabetes. These results were supported in phase II trials in T2DM patients with HbA_1c_ reductions of 2%, highly impressive body weight reductions of 5-10% (max 11.3 kg) and significant reductions in waist-circumference demonstrated following 12 weeks treatment (297). Moreover, comparison to the established GLP-1R mimetic, dulaglutide, proved tirzepatide to elicit more significant reductions in body weight (-4.52 kg/6.4% compared to -1.3 kg/1.8% for dulaglutide after 4 weeks), with the authors concluding inclusion of GIPR agonism builds upon sole GLP-1R activation to enhance weight loss *via* modulating appetite and gastric emptying, with the antiemetic effect of GIPR also improving tolerability (296). It is likely further mechanistic investigation will be pursued to fully elucidate the biological processes at play, especially as no single effect could be entirely attributed to GIPR/GLP-1R agonism (318). Thus, while important synergy is likely to be occurring, confirmation is required.

Tirzepatide has now progressed to phase III clinical trials in T2DM, and we await data from these studies with great anticipation. In similar fashion to the GLP-1/glucagon analogues discussed, tirzepatide has also found application in the treatment of NASH, with a follow-on study in T2DM patients revealing that several biomarkers of liver inflammation were reduced in patients receiving higher doses of the analogue (319). Indeed, a number of other analogues such as NN9389 (GIP and semaglutide combination), CT-868 and TAK-094 are all currently in phase I clinical trials as potential T2DM treatments (298), but any detailed literature on these analogues remains elusive at the time of writing.

### Dual Agonism With GLP-1 and OtherGut Peptides

A literature search for dual agonists also reveals some slightly left-field combinations with GLP-1, although importantly these involve other gut hormones shown to be upregulated by bariatric surgery (286). The combinations explored so far in preclinical studies all have the potential to elicit a range of additional effects on various systems in the body (**Figure 3**). For example, a long-acting GLP-1/CCK hybrid peptide has been developed which employs the key regions of (pGlu-Gln)-CCK-8, a stabilised form of CCK (320), and exendin-4 attached to one another *via* a linker molecule (**Table 5**). Through simultaneous activation of both GLP-1 and CCK-2 receptors, this co-agonist outperformed exendin-4 in terms of satiety and body weight reductions in obese-diabetic mice (299). A similar molecule, essentially reversing the configuration of GLP-1 and CCK components [(300); **Table 5**], also highlights the potential of dual receptor activation in this regard, outperforming (pGlu-Gln)-CCK-8 in terms of body weight reduction following 10 weeks treatment in DIO mice (300).

A GLP-1/secretin chimeric peptide, based on the sequence of secretin with GLP-1R activity induced *via* substitution of important GLP-1 residues (**Table 5**), has been developed. This peptide decreased food intake and body weight more effectively than liraglutide alone (301). Moreover, this analogue improved short-term glycaemic control (39% fall in fasting blood glucose), HbA_1c_ (-1.6%) and promoted a 78% rise in beta cell mass following twice daily s.c. administration over an 8 week period in diabetic, *db/db* mice (301).

Another successful, but seemingly counterintuitive pairing is the combination of GLP-1 and PYY. PYY is insulinostatic but holds therapeutic potential due to induction of beta cell rest, promotion of beta-cell mass, satiety and weight loss (321). Moreover, a synergistic effect between PYY and GLP-1 has been established (322), supporting their incorporation in a co-agonist. One such peptide, termed EP45, has been developed as a chimeric peptide employing PYY (25–36) incorporated with exendin-4(1–33); **Table 5**). Indeed, the peptide was demonstrated to effectively activate both GLP-1R and NPY2R in transfected cell lines (302), but *in vivo* application is yet to be published.

Finally, an enzyme resistant GLP-1/xenin dual-agonist, Exendin-4/xenin-8-Gln (303), has been developed with xenin, which is a regulatory peptide co-secreted postprandially with GIP from intestinal K-cells [(323); **Table 5**]. Xenin is known to potentiate the actions of GIP (324), and in addition to positive glycaemic outcomes, through reduced appetite and augmented insulin secretion, the peptide also restored GIP sensitivity (303) that is dampened in obesity (325). Consistent with these actions, Exendin-4/xenin-8-Gln induced substantial benefits in DIO diabetic mice (303).

### Conjugation of GLP-1 and Nuclear Hormones

Beyond the incorporation of GLP-1 with other gut hormones, there is also growing interest concerning the conjugation of GLP-1 with nuclear hormones like oestrogen, thyroid hormone (T_3_) and dexamethasone (326). In particular, the conjugation of GLP-1-estrogen allows selective targeting of oestrogen receptors (ER) in GLP-1R expressing cells. This reduces obesity and improves dyslipidaemia and hyperglycaemia more so than sole activation of either GLP-1R or ER (327). In relation to the metabolic effects of these conjugates, preclinical studies in rodents have demonstrated that these conjugates act on reward centres within the supramammillary nucleus to induce an anorectic effect (328), which positively influences glycaemia. Moreover, such conjugates were demonstrated to improve beta-cell function and survival (329), which in a study employing a combination of GLP-1-estrogen and insulin in a DIO model of diabetes, allowed for a 60% reduction in the insulin dose compared to a control group of animals receiving insulin monotherapy (330). While conjugation to GLP-1 proves an effective method to prevent the oncogenic and gynaecological actions of oestrogen (331), distinct differences in the hormonal aetiologies of obesity in males and females have demonstrated that administration of such agents in different sexes of mice elicit subtle differences in obesity-related inflammation pathways (332, 333). Thus, the impact of gender in relation to the applicability of these agents needs to be further explored.

### Triple Agonism With GLP-1

Given the successful development of GLP-1/GIP and GLP-1/glucagon dual-agonists (293, 296), the next obvious step was to develop triple-agonists based on these three gut hormones [(318); **Table 5**]. One such molecule, termed YAG-glucagon (**Table 5**), is an analogue based on human glucagon with a number of amino acid substitutions to impart GIPR and GLP-1R agonism (304). The DPP-4 resistant analogue was demonstrated to be an effective tri-agonist *in vitro*, while twice-daily administration in DIO mice manifested in improved blood glucose, circulating insulin and enhanced insulin sensitivity (304). While this molecule has not surpassed preclinical stage, a couple of examples appear to be progressing well at present. LY3437943, a reported tri-agonist is currently undergoing phase I trials in management of obesity-diabetes (307). More data is available for HM15211, a tri-agonist employing a GLP-1/GIP/glucagon peptide (sequence not available) attached to a human aglycosylate Fc fragment which prolongs half-life to permit once weekly administration [(334); **Table 5**]. HM15211was more effective than daily administration of liraglutide in increasing energy expenditure, with improvements in weight loss and hepatic inflammation markers in rodent models (334), and is currently recruiting for phase II trials as a treatment for NASH (308).

Similar to single GLP-1 agonists, a tri-agonist termed TA is also finding application with regards to neuroprotection [(309); **Table 5**]. This hybridised GLP-1/GIP/glucagon activator was initially developed for management of obesity-diabetes and showed promising preclinical results in rodent models of diabetes-obesity (310). However, the more recent repurposing of this molecule towards management of AD is particularly exciting, with daily administration of the analogue in a murine model of AD over a 2 month period reversing memory deficit, reducing pro-mitochondrial apoptosis markers and upregulating growth factors involved in synaptic function (309). The preclinical study has not been followed-up to date, but represents a potentially fruitful new avenue for the application of PGDP-based multiagonists.

Interestingly, the aforementioned GLP-1/xenin combination has been exploited further with the development of the triple-agonist exendin‐4/gastrin/xenin‐8‐Gln (335), a direct descendent of the previously discussed dual-agonist (303). This incorporates the hexapeptide gastrin into its sequence (**Table 5**), evoking the ability to agonise GLP-1R, CCK2R and NTSR1 in tandem (335). Preclinical studies with this peptide were promising, eliciting improved glycaemic control when administered twice daily in DIO diabetic mice over 21 days, through elevation in circulating insulin levels, improved insulin and GIP sensitivity, with encouraging reductions in fat mass, triglycerides and cholesterol levels (335). Moreover, this analogue has been further modified *via* the covalent attachment of a hexadecanoyl fatty acid to improve circulating half-life and duration of effect (**Table 5**), with twice daily administration in obese-diabetic *ob/ob* mice recapitulating the metabolic benefits attained with the non-acylated form (305).

## Other Possible Glucagon Therapeutics

The recognised role of hyperglucagonaemia in the pathophysiology of diabetes, and the effectiveness of concomitant activation of GCGR, alongside GLP-1R by oxyntomodulin, raises an apparently conflicting question: can glucagon antagonists or glucagon agonists be utilised as a diabetes or obesity therapy? A similar question exists for therapeutic GIP analogues (306). In fact, both aspects are being explored although neither is, as yet, fully understood, or nearing final stages of development.

### Glucagon Antagonists

The therapeutic potential for glucagon suppression is clear, especially given that a synthetic analogue of the glucagon suppressing hormone amylin, termed “Pramlinitide”, is currently prescribed in the USA as an adjunct to insulin therapy (336). However, pramlintide is not a specific inhibitor of glucagon secretion, as it also is well known to slow the rate of gastric emptying and induce satiety. Thus, direct glucagon receptor antagonism may represent a more specific alternative in this regard. While it is true that many small-molecule glucagon antagonists exist (337–339), these have been discounted due to undesirable pharmacokinetic properties which led to rapid renal clearance and diminished effects (337, 340). Moreover, off-target safety concerns were present, including activation of peroxisome proliferator-activated receptor-delta (PPAR-δ) (337), a transcription factor which plays roles in inflammation and certain cancers (341). That said, a few small molecules such as Eli Lilly and Co’s GRA LY2409021 have made it as far as phase II trials (342). These demonstrated promising reductions in HbA_1c_ but were ultimately let down by undesirable side-effect profiles, often eliciting potentially dangerous elevations in liver enzymes (343). Hence, a view was taken that development of glucagon peptide-based antagonists could herald better tolerated compounds with improved pharmacokinetic and safety profiles.

Logical design of such compounds took the sequence of native glucagon (**Table 6**), modifying the structure, paying particular attention to previously identified important residues for GCGR agonism, namely N-terminal His^1^, Gly^4^ and Asp^9^ residues (349–353), whilst also ensuring that they are resistant to the actions of DPP-4 [(349); **Figure 2**]. Two such analogues termed desHis^1^Pro^4^Glu^9^(Lys^12^PAL)-glucagon and desHis^1^Pro^4^Glu^9^(Lys^30^PAL)-glucagon [(346); **Table 6**], employed simple amino acid substitutions at residues 4 and 9, while His^1^ was deleted to produce compounds with the potential to effectively block the receptor and palmitic acid (PAL) was attached *via* linker molecules to substituted Lys residues at differing positions (346), a means of prolonging circulating half-life (351). Indeed, both molecules, as well as their non-acylated counterparts, were shown to be resistant to DPP-4 (346, 353). The compounds possessed strong antagonist properties, dose-dependently reducing glucagon-mediated cAMP production and insulin secretion together with counteracting glucagon-mediated hyperglycaemia *in vivo* (346).

**Table 6:** Glucagon antagonist peptides for T2DM.

Peptide Name	AA Sequence	Development Stage	Reference
Native glucagon	HSQGTFTSDYSKYLDSRRAQDFVQWLMNT	N/A	(10)
desHis^1^Glu^9^-glucagon	SQGTFTSEYSKYLDSRRAQDFVQWLMNT	Preclinical	(344, 345)
desHis^1^Pro^4^Glu^9^(Lys^12^PAL)-glucagon	SQPTFTSEYSK(*PAL)YLDSRRAQDFVQWLMNT	Preclinical	(346, 347)
desHis^1^Pro^4^Glu^9^(Lys^30^PAL)-glucagon	SQPTFTSEYSKYLDSRRAQDFVQWLMNTK(*PAL)	Preclinical	(345, 346, 348)
desHis^1^Glu^9^-glucagon-[mPEG]	SQGTFTSEYSKYLDSRRAQDFVQWLMNT-[mPEG]	Preclinical	(346)

Amino acid sequences are provided in their single-letter abbreviation format. Modifications from native sequences are highlighted by red lettering. Current development stages are provided for each. “mPEG” indicates mini-polyethylene glycol addition. “PAL” indicates the addition of a palmitic fatty acid chain.

Further related analogue development resulted in synthesis and characterisation of desHis^1^Glu^9^(Lys^30^PAL)-glucagon and desHis^1^Glu^9^-glucagon-[mPEG] [(299, 354); **Table 6**]. These peptides were resistant to DPP-4 degradation (344, 348, 355) and lacked adverse metabolic or islet morphological effects when administered twice daily to lean mice (347). Preclinical testing of desHis^1^Glu^9^-glucagon and desHis^1^Glu^9^(Lys^30^PAL)-glucagon in HFF obese mice reversed obesity-driven hyperinsulinaemia and insulin resistance together with improvements in lipid profile, glucose tolerance and increased pancreatic insulin stores (345).

These studies are typical of others that have led to development of peptidergic glucagon receptor antagonists for T2DM, in particular the first reported antagonistic, glucagon analogue [l-N alphatrinitrophenylhistidine, 12-homoarginine]-glucagon, which elicited decreases in circulating glucose of up to 65% with continuous infusion in anaesthetised rats (356). However, despite this preclinical promise peptide-based agents were largely abandoned at this point possibly due to short half-life in pursuit of small-molecule antagonists (357).

To date, no glucagon antagonist has reached regulatory approval, with previous safety concerns raised over hypoglycaemia (358), unfavourable alterations in serum lipid levels and liver enzymes (359, 360), as well as the potential for malignant hyperplasia of alpha-cells (361). Intriguingly, a similar tale is true for GLP-1R agonists, with a number of small molecule examples dotted through the literature (362), however none have managed to recapitulate the success of peptidergic agents. Preclinical data with peptide-based antagonists indicate more favourable side-effect profiles than small-molecules (345, 347). Thus, while work continues on small-molecule antagonists, such as RVT-1502, which has recently progressed through phase II trials, demonstrating reductions in HbA_1c_ of up to 1% over 12 weeks treatment, concerns over liver function still remain, and the compound has not ascended to phase III trials (363). Such concerns may lead to an upsurge in interest for peptide-based glucagon antagonists.

### Glucagon Agonists

Given the use of glucagon to rescue severe insulin-induced hypoglycaemia T1DM (53) and its ascribed role in the hyperglycaemia of diabetes (35, 51), the concept of using glucagon agonists therapeutically initially seems illogical. However, the surprising effectiveness of dual or triple agonism indicates that weight loss and increased energy expenditure associated with GCGR agonism can be exploited when the hyperglycaemic actions of the hormone are counteracted by the incretins GLP-1 and/or GIP (357, 364).

Several approaches have been explored to generate such, potentially useful, enzyme-resistant GCGR agonists including (D-Ser^2^)glucagon, where a D-amino acid substitution has been employed to impart DPP-4 resistance more effectively than N-acetyl-glucagon [(365); **Table 7**]. Insulin-releasing activity was maintained, but when further modified to generate (D-Ser^2^)glucagon-exe, an analogue with the nine C-terminal amino acid residues of exendin(1–39) (**Table 7**), clear antidiabetic benefits were induced (365). Importantly, inclusion of the C-terminal nonapeptide from exendin(1–39) in this molecule imparts the ability to agonise GLP-1R as well as GCGR, as demonstrated by reduced effectiveness in GLP-1R KO mice (365). Thus, twice daily administration of (D-Ser^2^)glucagon-exe in HFF mice improved glucose tolerance, insulin sensitivity and islet morphology, while improvements in energy expenditure, O_2_ consumption and physical activity together with reduced food-intake led to decreased body weight and influenced glycaemic improvement (365).

**Table 7:** Glucagon and related peptide analogues at preclinical stage for T2DM.

Peptide Name	AA Sequence	Target Receptor	Reference
Native glucagon	HSQGTFTSDYSKYLDSRRAQDFVQWLMNT	CGCR	(10)
N-Acetyl-glucagon	Ac-HSQGTFTSDYSKYLDSRRAQDFVQWLMNT	GCGR	(365)
(D-Ser^2^)glucagon	HDSQGTFTSDYSKYLDSRRAQDFVQWLMNT	GCGR/GLP-1R	(365)
(D-Ser^2^)glucagon-exe	HDSQGTFTSDYSKYLDSRRAQDFVQWLMNTPSSGAPPPS	GCGR/GLP-1R	(365)
Dogfish Glucagon	HSEGTFTSDYSKYMDNRRAKDFVQWLMSTKRNG	GCGR/GLP-1R	(366, 367)
(D-Ala^2^)dogfish glucagon	HDAEGTFTSDYSKYMDNRRAKDFVQWLMSTKRNG	GCGR/GLP-1R	(366, 367)
(D-Ala^2^)dogfish glucagon-exendin-4(31-39)	HDAEGTFTSDYSKYMDNRRAKDFVQWLMSTKRNGPSSGAPPPS	GCGR/GLP-1R	(366, 367)
(D-Ala^2^)dogfish glucagon-Lys^30^-γ-glutamyl-PAL	HDAEGTFTSDYSKYMDNRRAKDFVQWLMSTK(*PAL)RNG	GCGR/GLP-1R	(366, 367)
Paddlefish glucagon	HSQGMFTNDYSKYLEEKRAKEFVEWLKNGKS	GCGR/GLP-1R	(248)

Amino acid sequences are provided in their single-letter abbreviation format. Modifications from native sequences are highlighted by red lettering. The receptor targets for each molecule are provided. A “D” prefix before a residue indicates inclusion of the enantiomer for the naturally-occurring L form of the residue. “Ac” represents an N-terminal acetylation. “mPEG” indicates mini-polyethylene glycol addition. “PAL” indicates the addition of a palmitic fatty acid chain.

A number of naturally occurring, piscine-derived, glucagon peptides such as dogfish glucagon (and its analogues) and paddlefish glucagon have also been shown to possess potent antidiabetic/anti-obesity potential in cellular and animal models of diabetes [(248, 366, 367); **Table 7**]. Furthermore, studies using GLP1-R KO mice and cell lines indicated that benefits on glucose tolerance, beta-cell function, insulin sensitivity and circulating triglycerides were mediated *via* dual GCGR and GLP-1R agonism (248, 366). Thus, in relation to management of T2DM, inclusion of GCGR agonist in multiagonist molecules appears to be where the future novelty lies for such agents.

## Concluding Remarks

The application of proglucagon-derived peptides (PGDPs) in the management of conditions such as T2DM represents the pinnacle of a remarkable story in peptide discovery and rational drug design. It was shear perseverance which led to the elucidation of proglucagon almost six decades after that of glucagon (9, 11–16). Rapid discoveries followed of GLP-1 and GLP-2 (15, 16), both of which have been successfully exploited by peptide chemistry approaches to generate fully approved medications. While innovation has witnessed the production of increasingly long-acting agents, multi-action unimolecular agonists and novel delivery methods (67, 68, 70, 73–75, 195–197), there is still a growing need for ever more effective agents to counter obesity, diabetes and a host of other degenerative diseases. Better understanding of the physiology of PGDPs and their various roles in the likes of cognition, bone turnover, cardiovascular function, fertility and liver function (157, 174, 185, 334, 368, 369), may herald important future uses for proglucagon-derived therapeutics.

## Author Contributions

All authors contributed to the article and approved the submitted version.

## Conflict of Interest

PF, VG, NI,and FO’H are named on patents filed by Ulster University for the exploitation of incretin-based drugs and other peptide therapeutics.

The remaining authors declare that the research was conducted in the absence of any commercial or financial relationships that could be construed as a potential conflict of interest.
